# Binding of sFRP-3 to EGF in the Extra-Cellular Space Affects Proliferation, Differentiation and Morphogenetic Events Regulated by the Two Molecules

**DOI:** 10.1371/journal.pone.0002471

**Published:** 2008-06-18

**Authors:** Raffaella Scardigli, Cesare Gargioli, Daniela Tosoni, Ugo Borello, Maurilio Sampaolesi, Clara Sciorati, Stefano Cannata, Emilio Clementi, Silvia Brunelli, Giulio Cossu

**Affiliations:** 1 Department of Developmental Biology, Institute of Cell Biology and Tissue Engineering, San Raffaele Biomedical Science Park of Rome, Rome, Italy; 2 Department of Histology and Medical Embryology, II° Medical School, University of Rome “La Sapienza”, Rome, Italy; 3 Stem Cell Research Institute, H. “S. Raffaele”, Milan, Italy; 4 Department of Experimental Medicine, University of Pavia, Pavia, Italy; 5 Interdepartemental Stem Cell Research Institute, University Hospital Gasthuisberg, Leuven, Belgium; 6 Department of Biology, University of Tor Vergata, Rome, Italy; 7 Department of Preclinical Sciences, University of Milan, and E. Medea Scientific Institute, Milan, Italy; 8 Department of Experimental Medicine, University of Milan-Bicocca, Monza (Milan), Italy; 9 Department of Biology, University of Milan, Milan, Italy; University of Giessen Lung Center, Germany

## Abstract

**Background:**

sFRP-3 is a soluble antagonist of Wnts, widely expressed in developing embryos. The Wnt gene family comprises cysteine-rich secreted ligands that regulate cell proliferation, differentiation, organogenesis and oncogenesis of different organisms ranging from worms to mammals. In the canonical signal transduction pathway Wnt proteins bind to the extracellular domain of Frizzled receptors and consequently recruit Dishevelled (Dsh) to the cell membrane. In addition to Wnt membrane receptors belonging to the Frizzled family, several other molecules have been described which share homology in the CRD domain and lack the putative trans-membrane domain, such as sFRP molecules (soluble Frizzled Related Protein). Among them, sFRP-3 was originally isolated from bovine articular cartilage and also as a component of the Spemann organizer. sFRP-3 blocks Wnt-8 induced axis duplication in Xenopus embryos and binds to the surface of cells expressing a membrane-anchored form of Wnt-1. Injection of sFRP-3 mRNA blocks expression of XMyoD mRNA and leads to embryos with enlarged heads and shortened trunks.

**Methodology/Principal Findings:**

Here we report that sFRP-3 specifically blocks EGF-induced fibroblast proliferation and foci formation. Over-expression of sFRP-3 reverts EGF-mediated inhibition of hair follicle development in the mouse ectoderm while its ablation in Xenopus maintains EGF-mediated inhibition of ectoderm differentiation. Conversely, over-expression of EGF reverts the inhibition of somitic myogenesis and axis truncation in Xenopus and mouse embryos caused by sFRP-3. *In vitro* experiments demonstrated a direct binding of EGF to sFRP-3 both on heparin and on the surface of CHO cells where the molecule had been membrane anchored.

**Conclusions/Significance:**

sFRP-3 and EGF reciprocally inhibit their effects on cell proliferation, differentiation and morphogenesis and indeed are expressed in contiguous domains of the embryo, suggesting that in addition to their canonical ligands (Wnt and EGF receptor, respectively) these molecules bind to each other and regulate their activities during embryogenesis.

## Introduction

The *Wnt* gene family comprises cysteine-rich secreted ligands that regulate cell proliferation, differentiation, organogenesis and oncogenesis of different organisms ranging from worms to mammals [1], [2]. In the canonical pathway Wnts bind to the extracellular domain of Frizzled receptors [3], [4] and through canonical β-catenin or Ca^2+^/PKC dependent pathways activates Wnt target genes [5]. In addition to Frizzled receptors, four sFRP (Soluble Frizzled Related Protein), sharing homology in the CRD domain but lacking the putative trans-membrane domain have been identified [6]. *sFRP-3*, also known as *Frzb-1*, was originally isolated from bovine articular cartilage and also as a component of the Spemann organizer [7]. sFRP-3 binds to the surface of cells expressing a membrane-anchored form of Wnt-1 and blocks Wnt-8 induced axis duplication in Xenopus embryos [8]. Injection of sFRP-3 mRNA blocks expression of XMyoD mRNA and leads to embryos with enlarged heads and shortened trunks [8].

Recently, unforeseen cross-talks among different signaling pathways have been shown to affect several cellular responses both in embryogenesis and in oncogenesis. These interactions occur both in the extra-cellular space (ligand-ligand interaction) at the level of membrane receptors, and in the cytoplasm by second messenger molecules, such as SMAD, that are shared by different signaling pathways. In Drosophila, it is well known that Wg and EGF together regulate several morphogenetic events such as imaginal disc formation [9]–[11]. Whether this implies physical interaction among the signaling molecules or members of their signaling cascades is still unknown.

Here we show reciprocal inhibition of many biological activities exerted by EGF and sFRP-3 that are expressed in contiguous domains of both mesoderm and neuroectoderm and bind to each other *in vitro*. Thus one cross-talk between the Wnt and the EGF pathways is at the level of ligand binding in the extracellular space and may regulate reciprocal activities during embryogenesis.

## Results

### sFRP-3 expression alters C3H10T1/2 fibroblasts morphology and proliferation

**Figure 1:**
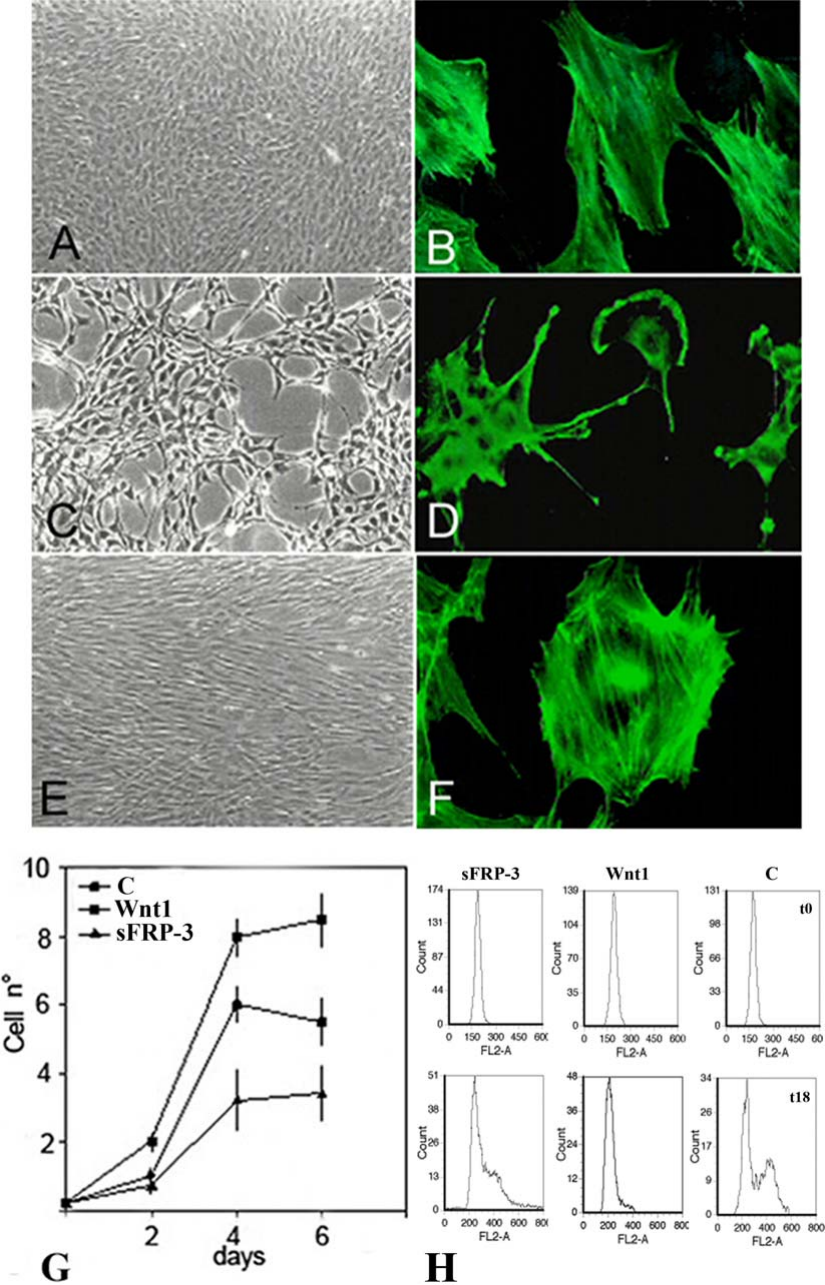
sFRP-3 modifies cytoskeleton and reduces proliferation of C3H10T1/2 fibroblast. Morphology of C3H10T1/2 cells infected with vectors expressing puro cDNA (A), sFRP-3 cDNA (C), Wnt-1 cDNA (E). When stained for F-actin with falloidin (see Materials and Methods) both control cells (B) and Wnt-1 expressing cells (F) showed well organize stress fibers, whereas sFRP-3 expressing cells (D) showed membrane ruffles, filopodia and disorganized actin fibers. (G) Growth curves for control, Wnt-1 and sFRP-3 expressing cells. Cells were plated at an initial density of 2×10^4^ cells/60 mm dish in DMEM 5% FCS and re-fed on day 2 and 4. Cell numbers were measured by detaching cells with trypsin and counting triplicate samples for each point. (H) C3H10T1/2 cells stably transfected with sFRP-3, Wnt1 expression vector or empty vector as control (C), maintained in DMEM 10% FCS, were starved in DMEM serum free medium. After 24 serum was added again to the medium and cells analyzed for their DNA content by flow cytometry after Propidium Iodide, staining after 0, 18 and 20 h. Representative plots are shown in the upper panel for the three cell lines at t0 (upper plots) and t18 (lower plots).

When retrovirally over-expressed in C3H10T1/2 cells, that do not express Wnts or sFRPs (see next paragraph for details), sFRP-3 affects their morphology and inhibits their proliferation. As shown in Figure 1, cells expressing sFRP-3 appeared smaller than their parental counterparts and projected several cytoplasm processes (compare 1C with 1A). In addition sFRP-3 expression altered the cytoskeleton, inducing stress fiber disorganization, membrane ruffling, and filopodia (compare 1D with 1B and 1F). In contrast, Wnt-1-over-expressing cells grew more densely as a monolayer, forming bundles lined up in uniform directions (Fig. 1E). As previously reported [12] Wnt-1-expressing cells proliferated faster than parental cells, with an almost two-fold increase in plateau cell density, while sFRP-3-expressing cells proliferated more slowly showing a reduced plateau cell density (Fig. 1G). Flow cytometry analysis revealed a significant accumulation of sFRP-3-expressing cells in G0/G1 phase in comparison with control cells, while Wnt-1-expressing cells exhibited a significant reduction of cells in the G0/G1 phase (Fig. 1H; see quantification of these data in Table S1).

Conditioned medium from sFRP-3-expressing, but not from control cells, inhibited proliferation of Wnt-1 expressing but also of control C3H10T1/2 fibroblasts (not shown).

### sFRP-3 specifically inhibits EGF mediated proliferation of C3H10T1/2 cells

**Figure 2:**
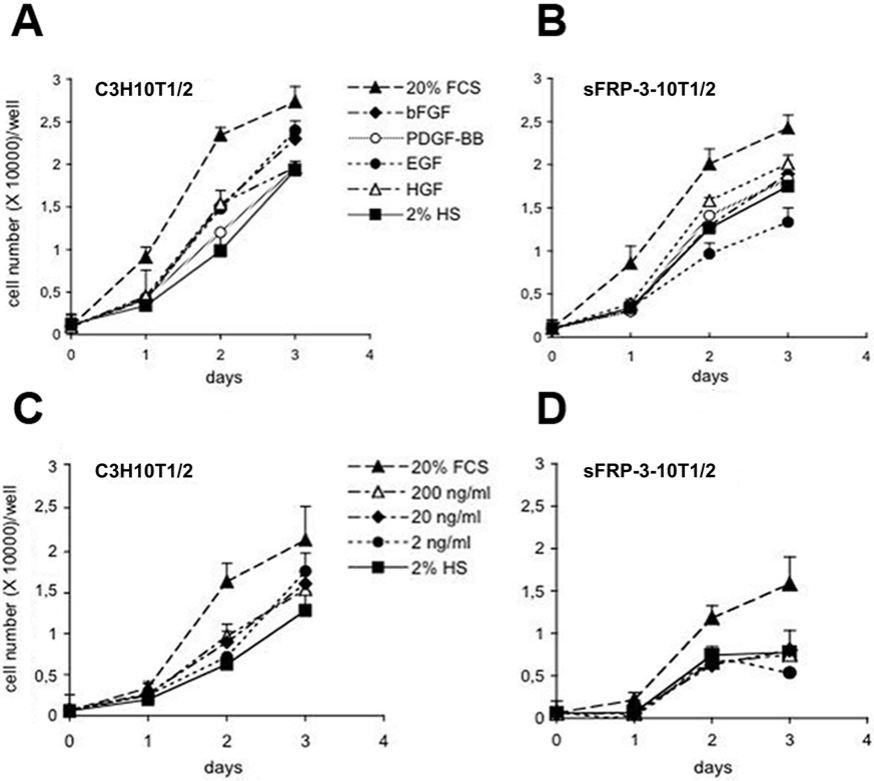
sFRP-3 inhibits proliferation induced by EGF. (A–D) Effect of growth factors on sFRP-3 expressing C3H10T1/2 cells. Growth curves of C3H10T1/2 cells (A) and sFRP-3 expressing C3H10T1/2 (B) cultivated in 2% horse serum (HS)±specific growth factors. Cells grown in 20% FCS or 2% HS alone represented positive and negative control respectively. At the time points indicated, cells were fixed and stained with 0.1% crystal violet solution as described in the Materials and Methods. Dose-dependent effect of EGF on control (C) and sFRP-3 expressing C3H10T1/2 cells (D).

C3H10T1/2 cells express few Wnts such as Wnt2, 2b, 4, 5, 5b, and 9a at extremely low, basically background, level (0,0001% of reference housekeeping genes, see Table S2) as demonstrated by quantitative RT-PCR experiments performed on Wnt-pathway PCR array (SuperArray Bioscience Corporation, see Materials and Methods for details). Thus, sFRP-3 dependent inhibition of C3H10T1/2 cells proliferation is not likely due to the inhibition of a Wnts-mediated autocrine loop, also because other necessary molecules of the Wnt pathway, such as Lef1, are not expressed in these cells (Table S2). Since proliferation depends upon the action of different mitogens present in the fetal calf serum (FCS), such as EGF, FGF, PDGF, we investigated whether sFRP-3 could block the mitogenic response mediated by any of these factors. To test this hypothesis we exposed serum starved control and sFRP-3 expressing C3H10T1/2 cells to EGF (100 ng/ml), bFGF (10 ng/ml), PDGF (0.4 nM) or HGF (20 ng/ml). As expected, control C3H10T1/2 cells proliferate in response to any of these growth factors, even though to a different extent (Fig. 2A). Interestingly, sFRP-3 expressing cells were able to proliferate in response to all growth factors like control cells, with the notable exception of EGF (Fig. 2B), in presence of which they failed to proliferate, even when present at the highest concentration (Fig. 2C–D). This data clearly demonstrates that reduced proliferation of sFRP-3 expressing C3H10T1/2 cells in high serum depends by the inability of these cells to respond to serum EGF, and suggests a possible direct interaction between sFRP-3 and EGF.

### sFRP-3 inhibits the EGF-induced colony formation and focal transformation of EGF-receptor over-expressing NIH 3T3 cells

To further investigate this unexpected finding, we assessed whether other known biological activities of EGF might be blocked by sFRP-3. We tested: 1) EGF induced oncogenic phenotype in transformed cells; 2) EGF triggered transient increases in [Ca^2+^]_i_ in 3T3; 3) EGF inhibition of hair follicle appearance in explants of embryonic ectoderm.

**Figure 3:**
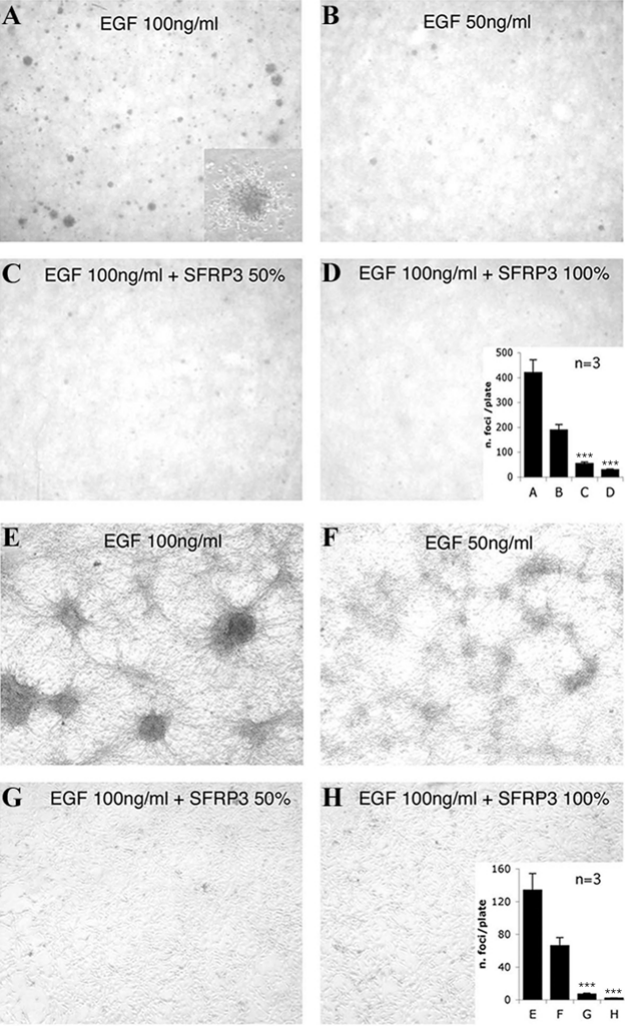
sFRP-3 inhibits proliferation EGF-dependent foci formation. (A–D) sFRP-3 inhibits the EGF-induced colony formation and focal transformation of EGF-receptor over-expressing NIH 3T3 cells. EGF-dependent cell growth in soft agar. NIH3T3 clone 17 cells suspended in agar containing normal medium and treated with either 100 ng/ml (A) or 50 ng/ml (B) of EGF formed clearly visible colonies. The effect of EGF was inhibited in cells suspended in agar containing a 1∶1 mixture of normal medium and sFRP-3-containing medium (CM) (C) or the sFRP-3-CM alone (D). Photographs were taken after 20 days of growth at 2× magnification. Insert in D shows a 20× magnification of one colony; insert in H shows the results obtained by counting the colonies in 3 independent, reproducible experiments±SE. Error bars represent s.e.m. Triple asterisks, P<0.001 vs EGF. (E–H) EGF-dependent focal transformation. NIH3T3 clone 17 cells cultured for 10 days in normal medium containing 100 ng/ml EGF (E) or 50 ng/ml EGF (F) formed foci. This focal transformation was inhibited incubating the cells in a 1∶1 mixture of normal medium and sFRP-3-CM (G) or the sFRP-3-CM alone (H). Photographs were taken after 10 days of growth at 10× magnification. Insert in H shows the results obtained by counting foci in 3 independent, reproducible experiments±SE. Error bars represent s.e.m. Triple asterisks, P<0.001 vs EGF.

A clone (n°17) of NIH 3T3 cells that over-expresses the EGF receptor [13], [14] undergoes EGF-dependent focal transformation and generates colonies in soft agar in the presence of EGF. As expected, twenty days after plating, EGF induced the formation of clearly visible colonies in agar, in a concentration-dependent way (Fig. 3A, B). This effect was inhibited by incubation of cells in sFRP-3-containing agar (Fig. 3C, D). The grade of inhibition correlated with the percentage of sFRP-3 containing medium used for agar preparation. The inhibitory effect of sFRP-3 was also evaluated by assessing EGF-dependent focal transformation (Fig. 3E–H). EGF induced a concentration-dependent formation of foci (Fig. 3E, F) that was inhibited by incubation of cells in the presence of sFRP-3 containing medium (Fig. 3G, H).

### sFRP-3 specifically inhibits acute, EGF-induced changes in Ca2+ homeostasis

**Figure 4:**
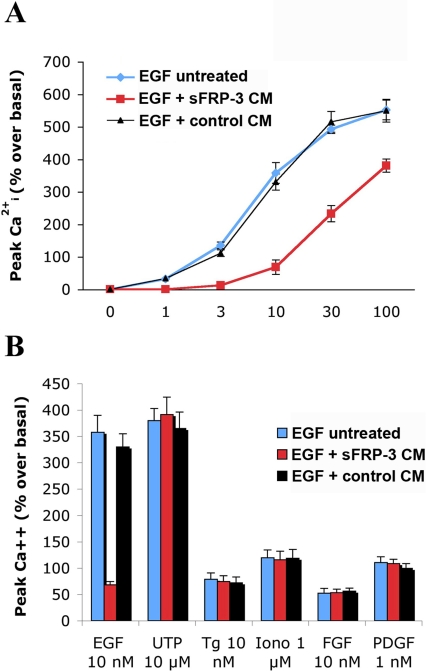
sFRP-3 inhibits the EGF-induced [Ca^2+^]_i_ variations in EGFR-T17 cells. Fura-2 loaded cells, suspended in KRH medium, were incubated with medium conditioned (CM) either by sFRP-3-expressing or not expressing cells, and challenged with increasing concentrations of EGF (A), or submaximal concentrations of FGF (10 nM), PDGF (1 nM), UTP (10 µM), thapsigargin (Tg, 10 nM) or iomomycin (Iono, 1 µM) as indicated (B). Shown are the [Ca^2+^]_i_ changes±s.e.m. measured as % increases over resting [Ca^2+^]_i_ values (n = 4). These were 136±12 nM and were not changed by the addition of sFRP-3 CM or control CM.

The above results suggest that sFRP-3 interferes with the EGF signaling pathway. To test whether the interaction may be direct, we investigated a rapid effect of EGF, i.e. increases in [Ca^2+^]_i_ that are transient and occur within seconds after growth factor administration [15]. T17 cells were loaded with the Ca^2+^-sensitive dye fura-2, and peak [Ca^2+^]_i_ variations induced by increasing concentrations of EGF in the presence of medium conditioned by either sFRP-3-expressing or not expressing (control) cells were recorded. As shown in Figure 4A, the medium conditioned by sFRP-3-expressing cells, but not the control medium, inhibited the EGF-induced [Ca^2+^]_i_ increases. These cells exhibit [Ca^2+^]_i_ responses also when stimulated with PDGF and FGF, the P2_Y_ receptor agonist UTP, the endo-sarcoplasmatic reticulum Ca^2+^ pump inhibitor thapsigargin or the Ca^2+^ ionophore ionomycin [15]: [Ca^2+^]_i_ increases induced by these molecules were not affected by sFRP-3 conditioned medium, indicating that the effect on the EGF evoked response is selective (Fig. 4B).

We then checked if sFRP-3 might affect EGF-induced phosphorylation of key signal transduction proteins *in vitro*. As shown in panel A of Figure S1, the addition of sFRP-3-CM to NIH-3T3 cells during EGF stimulation reduced both AKT and MAPK phosphorylation, further indicating that sFRP-3 interferes with EGF signaling pathway. This reduction was not observed in the case of bFGF stimulation (Fig. S1B).

### sFRP-3 reverts EGF-induced inhibition of hair follicle formation in the embryonic ectoderm

**Figure 5:**
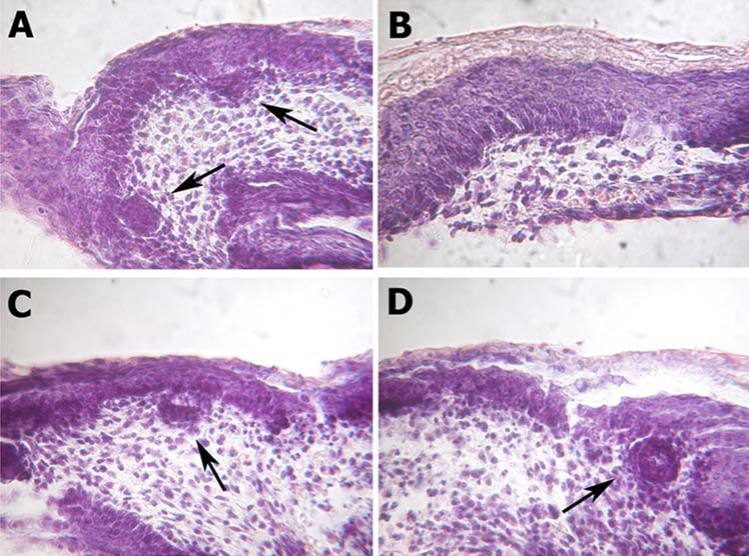
sFPR-3 and EGF reciprocal antagonism on hair follicle development. (A–D) sFPR-3 antagonizes the EGF effect rescuing the hair follicle formation in an organ culture of developing mouse skin. Ectoderm explants growth for 48 hours in control medium (A) or in control medium supplied with 7 µg/ml sFRP-3-HA (C), showing normal hair follicles differentiation (black arrows). (B) Ectoderm explants growth in organ culture in medium supplied with 100 ng/ml recombinant EGF for 48 hours, lacking hair follicle differentiation. (D) The co-treatment with EGF and sFPR-3 rescue the hair follicle formation (black arrow).

We then tested the ability of sFRP-3 to counteract also one complex biological activity of EGF *in vivo*. In the embryonic ectoderm, hair follicles form at E14.5 dpc: this process also occurs in tissue explants *in vitro* and EGF is known to inhibit follicle appearance when added to the culture medium [16]. In ectoderm explants grown in control medium we observed the appearance of numerous hair follicles (arrows in Fig. 5A). This was not the case in the presence of EGF (Fig. 5B). sFRP-3 alone did not influence follicle appearance (Fig. 5C) but it reverted EGF-induced inhibition of hair follicle (Fig. 5D) so that the number of follicles that formed in the presence of both molecules was similar to that formed in medium alone.

These data clearly demonstrate that sFRP-3 blocks all the biological actions of EGF on proliferation, cell signaling, and morphogenesis that we have tested.

### EGF blocks specifically the effect of sFRP-3 in Xenopus embryos

**Figure 6:**
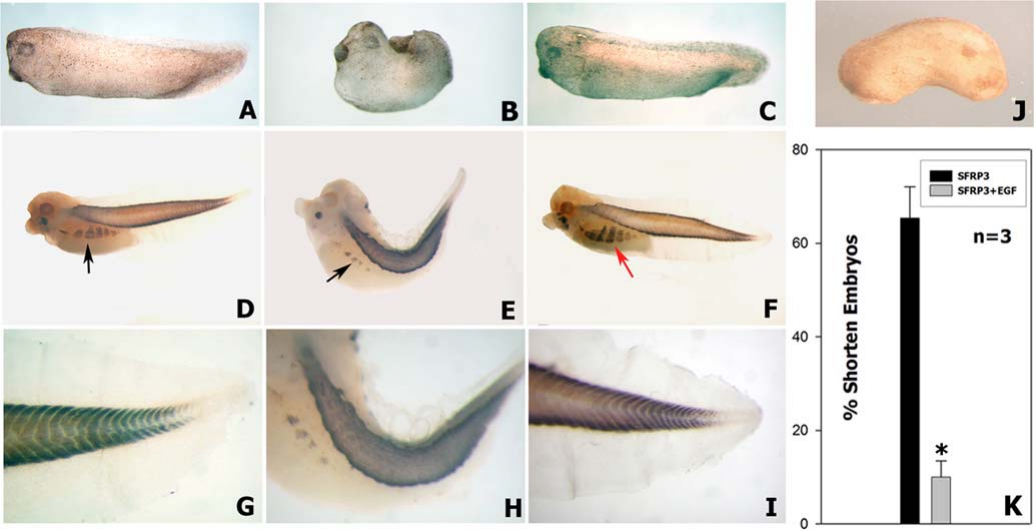
EGF specifically antagonizes sFPR-3 effects in Xenopus embryos. (A) Lateral view of a normal embryo at stage 28/29 NF un-injected. (B) Embryo injected with 4 ng of mouse sFPR-3 mRNA at stage 28/29 NF, showing a remarkable shortened of the trunk and the tail and a reduced muscle differentiation. (C) Rescue of the shortened phenotype by co-injection of 4 ng of sFPR-3 and 8 ng of EGF mouse mRNAs. (D–I) Whole mount immunostaining with 12–101 specific amphibians muscle monoclonal antibody, peroxidase detected. (D) Normal embryo un-injected at stage 35/36 NF, showing black labeling in the trunk and tail muscles and in the parietal muscle (black arrow). (E) Injected embryo with mouse sFPR-3 mRNA at stage 35/36, presenting a shortened trunk and a kinky tail; furthermore the parietal muscles (black arrow) are remarkably reduced. (F) Rescue of the shortened kinky phenotype, showing completely restored muscle differentiation also in the parietal muscle (red arrow). (G–I) High power magnification respectively of D–F, showing in detail the chevron like organization of the tail muscles of un-injected normal larvae (G), missing in the sFRP-3 treated tadpole tail (H) and restored in the co-injected larvae with EGF mouse mRNA (I). (J) Lateral view of a co-injected embryo with 4 ng of sFRP-3 and 8 ng of FGF-8 mouse coding mRNAs, showing no rescue of the sFRP-3 phenotype. (K) Quantization of multiple injections (n = 3) showing the rate of the phenotype rescue by co-injection with 8 ng of EGF mouse mRNA after the injection of sFPR-3 (asterisk indicates statistical significance P<0.001).

We then tested whether EGF may conversely interfere with the effects of sFRP-3. sFRP-3 mRNA injection in Xenopus embryos inhibits trunk elongation and skeletal muscle formation [8]. We first demonstrated that mouse sFRP-3 is able to induce the same effect as the Xenopus homolog (Fig. 6A, B). We then co-injected sFRP-3 and EGF mRNAs into four cell stage Xenopus embryos: the results of this experiment showed a reversion of sFRP3-induced phenotype in most of the co-injected embryos (Fig. 6C). Using a 2-fold excess of EGF mRNA concentration (4 ng sFRP-3 and 8 ng EGF) the rate of shortened embryos was significantly reduced (from 65,3% shortened embryos n = 149, to 9,9% n = 127, as shown in Fig. 6K). Staining of sFRP-3-injected embryos with anti-sarcomeric myosin antibody revealed a drastic reduction of the parietal muscles and disruption of the myotome pattern in comparison with control, un-injected embryos (Fig. 6D, E, G, H). However, co-injection of sFRP-3 and EGF mRNAs caused a recover of the chevron like organization of the trunk/tail skeletal muscle and fully restored parietal muscles differentiation (Fig. 6F and 6I).

In order to prove the specificity of this interaction we tested the ability of mFGF8 [17] to revert sFRP-3 phenotype. As reported [18], XeFGF mRNA injection induces embryo posteriorization (2 pg, data not shown), with a lack of anterior structure such as head and eyes. The same phenotype was observed when we co-inject mFGF-8 with sFRP-3 coding mRNAs (4 ng and 8 ng respectively), together with the sFRP-3-induced shortening of the embryo (Fig. 6J), indicating that mFGF-8 is not able to revert the sFRP-3 phenotype. Thus we can conclude that the recovery of shorten embryo injected by sFRP-3 mRNA is EGF specific. These results show that the interaction between sFRP-3 and EGF occurs also *in vivo* in Xenopus embryos.

### EGF reverts sFRP-3-induced inhibition of axis truncation and somitic myogenesis in the mouse embryo

**Figure 7:**
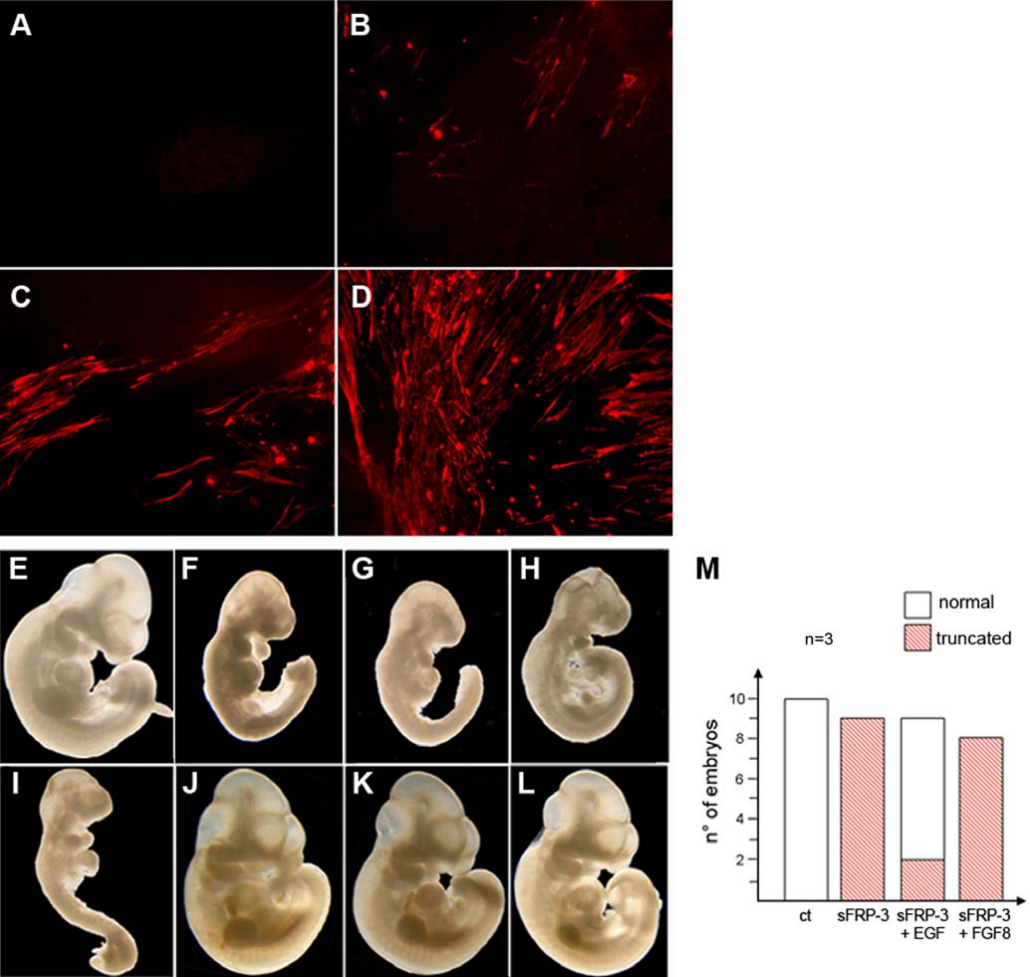
EGF reverts sFRP-3-induced inhibition of somitic myogenesis. (A–D) EGF reverts sFRP-3-induced inhibition of somitic myogenesis in explant cultures. MyHC antibody staining on explants cultures of presomitic mesoderm from E9.5 *Myf5a2* embryos in the presence of sFRP-3 and EGF proteins. Mesoderm differentiation, which is almost completely abolished in the presence of sFRP-3 protein (A), is partially to fully restored when increasing amount of EGF are added to the culture (B–D: 50, 100 and 200 ng/ml), as revealed by the increasing number of positive MyHC fibers. (E–M) EGF rescues inhibition of myogenesis induced by sFRP-3 in vivo. (F,G) Transplacental delivery of s-FRP3 causes severe malformations of the caudal region of injected embryos. (J–L) Normal embryo morphology is restored when sFRP-3 is co-injected together with EGF, but not with FGF (H; I). (E) Control not-injected embryo. (M) Quantification of injected embryos with their relative phenotype in 3 independent, reproducible experiments.

We previously showed that sFRP-3 reversibly inhibits myogenesis in explants of pre-somitic mesoderm of mouse embryos [19]. We therefore differentiated explant cultures of pre-somitic mesoderm (with its associated neural tube and surface ectoderm) in the presence of sFRP-3 conditioned medium and increasing concentration of EGF. Differentiated, myosin positive, muscle cells were observed in control explants (not shown) but not in explants cultured in the presence of sFRP-3 (Fig. 7A). EGF (which by itself had no effect: not shown) caused dose-dependent reappearance of differentiated, myosin positive myocytes (Fig. 7B–D). Thus EGF reverts sFRP-3-induced inhibition of somitic myogenesis. We had also shown [20] that trans-placental delivery of sFRP-3 caused an axis truncation in the mouse embryos similar to what observed in Xenopus, similarly inhibiting skeletal myogenesis. To test whether these effects could also be reverted by EGF, we performed transplacental delivery of the two proteins throughout simultaneous injection of WOP cells that stably expressed either sFRP-3 or EGF. As previously demonstrated, sFRP-3 delivered at the onset of the materno-fetal circulation induced a significant axis truncation (compare Fig. 7F, G with 7E) that what reversed when we co-injected WOP cells that express sFRP-3 together with WOP cells that express EGF: in such a case 75% of the embryos display normal morphology (Fig. 7J–L). Importantly, sFRP-3 phenotype could not be rescued by the injection of FGF8-expressing WOP cells (Fig. 7H, I), indicating that EGF reverts morphogenetic alterations induced by sFRP-3 in a specific manner, also *in vivo*. Similarly, *in vivo* inhibition of myogenesis by sFRP-3 was reverted by EGF but not by FGF8 (data not shown).

### EGF-mediated inhibition of sFRP-3 is Wnt-independent

**Figure 8:**
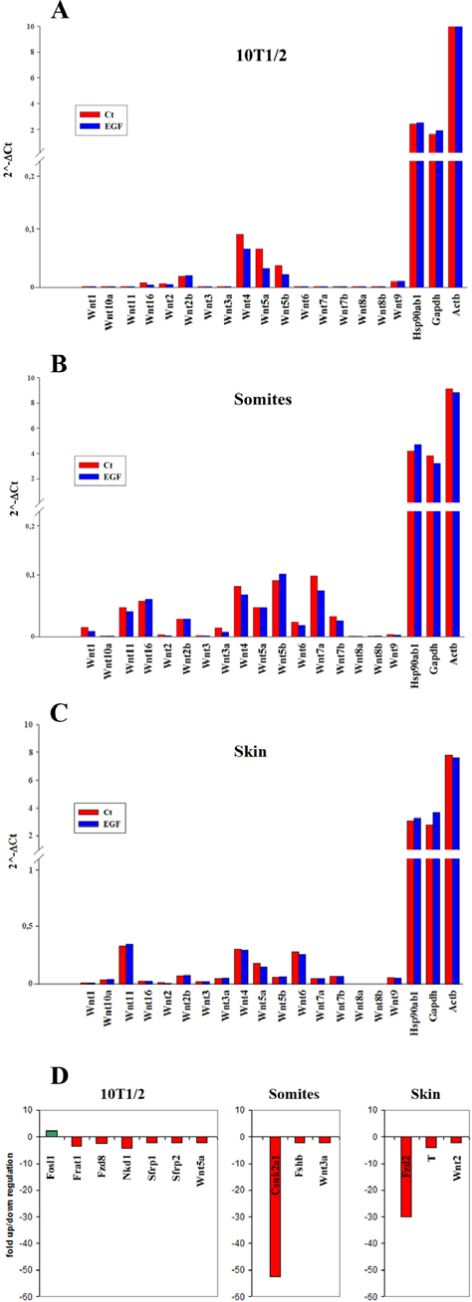
Wnts expression profile in C3H10T1/2 and embryonic explant cultures. Wnt genes expression in C3H10T1/2 fibroblast (panel A), somites (panel B) and embryonic skin (panel C). Histograms represent the relative expression level for each gene in control (red bars) and EGF-treated (blue bars) samples, as calculated with the 2^ˆ^ (−Δ Ct) formula in comparison with the housekeeping genes Hsp90ab1, Gapdh and Actb (see Materials and Methods for details). (Panel D). EGF-modulated genes in C3H10T1/2 fibroblast, somites and embryonic skin. For genes description, see Table S2.

As sFRP-3 is a known inhibitor of Wnts, the possibility exists that EGF blocks its activity by stimulating the expression of Wnts in target cells. To test this hypothesis, we analyzed the expression of Wnts in C3H10T1/2 cells, embryonic skin and somites explants cultured for 24 h *in vitro* in the presence of EGF. To this aim, we used a PCR array kit containing 84 Wnts and Wnt-pathway genes (SuperArray Bioscience Corporation, see Materials and Methods for details). All Wnt genes tested are expressed at very low level in C3H10T1/2 cells, skin and somites explants (Fig. 8, panels A, B, C). Moreover, none of the Wnt genes are induced upon 24 h of EGF stimulation. Indeed, very few genes of this family are modulated upon EGF stimulation, mostly in a down-regulation manner (Fig. 8D; see Table S2 for complete C3H10T1/2 array results, n/a for skin and somites explants). We can thus conclude that the EGF-mediated inhibition of sFRP-3 observed in vivo is Wnt-independent, suggesting a direct interaction between EGF and sFRP-3 molecules.

### sFRP-3 binds EGF *in vitro*

**Figure 9:**
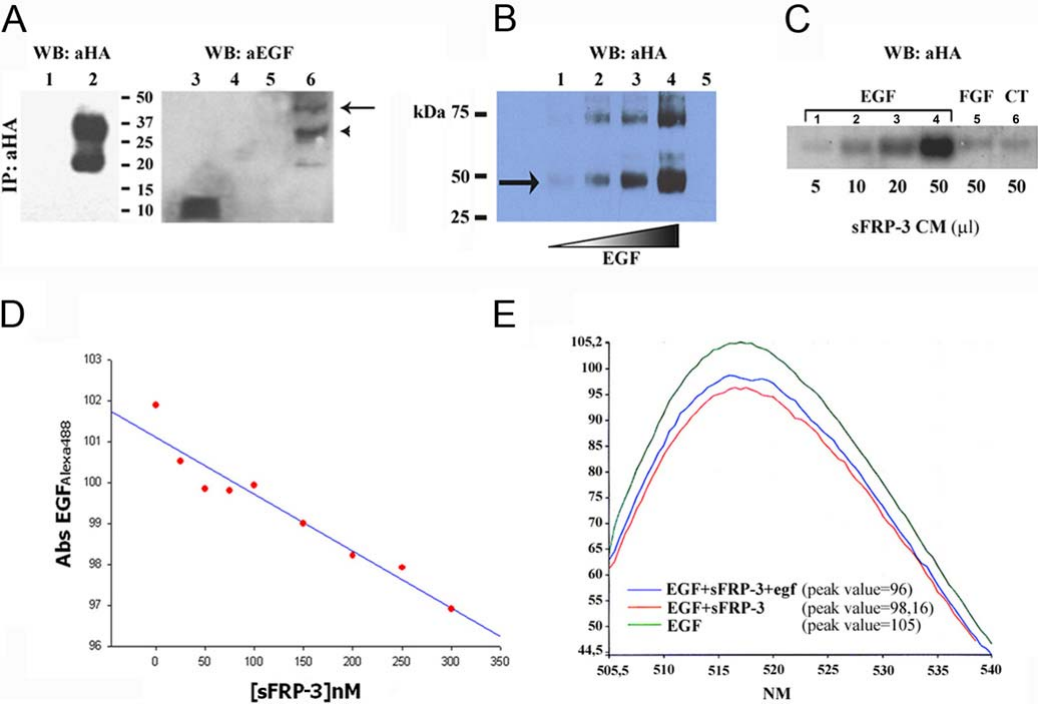
sFRP-3 binds EGF *in vitro*. (A) Co-IP of sFRP-3-HA and EGF. Supernatant from 293 cells transfected with control plasmid (lanes 1, 4) or purified sFRP-3-HA (lanes 2, 5, 6) were incubated with mouse recEGF (lanes 4, 6), DTSSP cross-linked and analysed under non-reducing conditions. Western blot analysis with anti-HA (lanes 1, 2) and anti-EGF antibodies (lanes 3–6) revealed the presence of EGF bound to sFRP-3-HA (lane 6), but not in the control sample (lane 4). Note that the shift in molecular weight of the band(s) corresponding to sFRP-HA (lane 2) is due to EGF binding (arrow and arrowhead in lane 6). Free mouse recEGF was used as positive control for the immunoblot (lane 3). (B) Dose-dependent binding of EGF to sFRP-3. *In vitro* binding of increasing amount of EGF (100 ng, 200 ng, 400 ng and 600 ng,) to sFRP-3-HA (100 ng), followed by DTSSP crosslinking and revealed by Western blot analysis with anti-HA. The amount of EGF/sFRP-3 complexes (lanes 1–4) correlates with the increasing amount of EGF. As control for binding specificity, 100 ng of sFRP-3-HA alone (lane 5, not detectable) were cross-linked and revealed as before. (C) *In vitro* binding of sFRP-3 to EGF-loaded heparin beads. Western blot analysis with anti-HA on proteins adsorbed on EGF-loaded (lanes 1–4), FGF8-loaded (lane 5) and control beads (lane 6), following incubation with increasing amount of sFRP-3-HA supernatant (5–50 µl). sFRP-3 binds specifically to EGF, in a dose dependent manner. (D) Binding affinity of EGF for sFRP-3. EGFAlexa488 fluorescent absorbance at emission peak (517 nm) decreases in relation of increasing amount of sFRP-3. (E) Competition assay. Absorbance scanning from emission value 505 nm to 540 nm of EGFAlexa488 (green curve). A decreasing of the emission peak (517 nm) is shown in relation to EGFAlexa488/sFRP-3 interaction (red curve). By adding native egf to EGFAlexa488 prior to the incubation with sFRP-3, the absorbance reduction of the emission peak was smaller (blue curve).

We tested the ability of sFRP-3 to bind EGF *in vitro* by performing co-immunoprecipitation (co-IP) experiments. For this purpose, sFRP-3-HA protein was first immunopurified with an anti-HA affinity matrix (Covance) from the conditioned medium (CM) of 293T cells transfected with sFRP-3-HA plasmid and then incubated with recombinant EGF, followed by the addition of a non-cleavable, cross-linking reagent (DTSSP). The putative cross-linked complexes were then analyzed by SDS-PAGE. As shown in Figure 9A, we detected a clear cross-linked sFRP-3-HA/EGF complex with anti-EGF antibody only when EGF was incubated with the sFRP-3-HA protein (lane 6). The molecular weight of this complex (arrow in panel A) corresponded to the expected mass of sFRP-3-HA protein (37 KDa, upper band in lane 2) plus EGF protein (6 KDa, lane 3). The presence of a second band of lower molecular weight in the immunopurification (20 KDa, lower band in lane 2) might correspond to a partial degradation of sFRP-3-HA protein. Notably, also this product forms a complex of higher molecular weight in the presence of EGF (lane 6, arrowhead in panel A), indicating that it is specifically recognized by the anti-EGF antibody. We next performed a direct *in vitro* binding experiment between EGF and purified sFRP-3 by incubating sFRP-3-HA or HA peptide with increasing amount of EGF. Following DTSSP cross-linking, the resulting complexes were analyzed by Western Blot with the anti-HA antibody. Figure 9B shows that EGF binds sFRP-3 directly *in vitro*, giving rise to the same sFRP-3-HA/EGF complex previously described (arrow in panel B, lanes 1–4). The amount of sFRP-3-HA/EGF complex increased with increased EGF concentration. We also detected a second complex of approximately 75 kDa which could be explained by the ability of sFRP-3 protein to form dimers, as previously described [21]. Similarly to the smaller one, the amount of this second complex appears to be EGF dose-dependent, suggesting that EGF could induce sFRP-3 dimerization. DTSSP cross-linking demonstrates *in vitro* short range interaction between molecules but does not mimic a natural embryonic environment. To address this issue, heparin beads (mimicking heparan sulfate) were loaded with EGF and then incubated with increasing amount of sFRP-3-HA conditioned medium. The presence of bound sFRP-3 was then analyzed by western blot as described before. Figure 9C shows that sFRP-3 binds specifically to EGF in a dose-dependent manner (lanes 1–4), while neither FGF8-loaded nor control beads are able to bind sFRP-3 (lane 5 and 6, respectively).

To better understand the nature of the two proteins interaction, we performed binding experiments using luminescence spectrometry. The EGF/sFRP-3 interaction was studied by measuring changes in the absorbance spectrum of Alexa488-conjugated EGF, which gives stable and reproducible absorbance spectrum [22], in the presence of increasing amount of recFRP3. Addition of sFRP-3 to EGFAlexa488 caused a significant change in the EGFAlexa488 spectrum, i.e. a decrease in the absorbance value, in particular at emission peak = 517 nm (Fig. 9D, E). Interestingly, this decrement appeared to be sFRP-3 dose-dependent, as it diminished at increasing amount of sFRP-3 (Fig. 9D). To test the specificity, we performed a competition assay by adding native EGF (at a 10 fold molar excess) to EGFAlexa488 prior to the addition of sFRP-3 (at a 4 fold molar excess). In these conditions, the EGFAlexa488 absorbance value decreased of approximately 10 units at emission peak in the presence of sFRP-3, whereas it diminished of 7 units when native EGF was added before sFRP-3 (Fig. 9E). This difference likely depends upon a competition between the two types of EGF molecules for sFRP-3 binding, supporting the specificity of EGF/sFRP-3 interaction.

### Membrane-anchored sFRP-3 confers EGF binding to CHO cells

**Figure 10:**
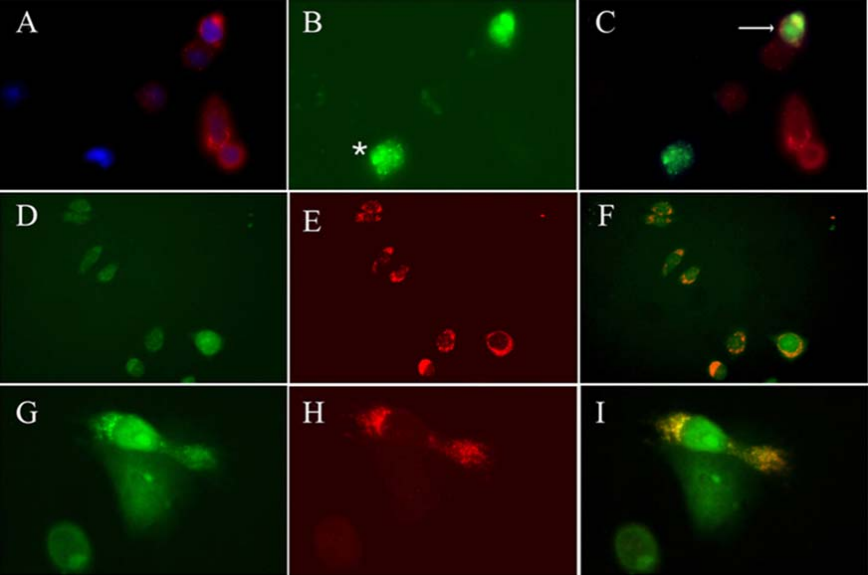
Membrane-anchored FRP-3 binds EGF at the cell surface. (A–C) Antibody staining for HA (in red, A,C) in CHO cells transfected with pCS2*FRP3-HACD4* shows FRP-3 expression on the cell surface. Upon administration of EGFAlexa488, EGF expression (green) is detectable only on whole intact CHO cell expressing FRP-3, but not on control cells (C). Star in panel B shows non-specific EGF binding on necrotic cell. Double labelling for HA and GFP shows co-localization of FRP-3 and EGF at the cell surface (arrow in C). (D–I) Anti-EGF antibody staining (in red) on FRP-3GFP CHO cells transfected with pTWEEN*FRP3-HACD4*. (D–F) Upon DTSSP cross-linking all CHO cells that express FRP-3CD4 (green staining in D) bind EGF at the membrane surface (red staining in E and merged colour in F). (G–I) 40× magnification of a single CHO cell that expressed FRP-3GFP (in green) and bind EGF (in red). Double labelling for GFP and anti-HA in panel I shows the two proteins interaction occurs at the membrane surface.

We finally tested the ability of sFRP-3 to bind EGF in the extra-cellular space. For this experiment, we generated a fusion protein in which FRP-3-HA was exposed to the extra-cellular compartment and anchored to the plasma membrane via the fused CD4 trans-membrane moiety. CHO cells, that lack EGF receptor [23], were transiently transfected either with pCS2*FRP3-HACD4* or pTWEEN*FRP3-HACD4* constructs that contain the *FRP3-HACD4* DNA sequence downstream the CMV promoter, or upstream the PGKEGFP cassette, respectively. Cell surface expression of the FRP3-HA- or FRP3-GFP- fusion protein was confirmed by immunofluorescence staining with anti-HA antibody (Fig. 10A, C) or GFP expression (Fig. 10D, F). Specific binding of EGF in the extra-cellular space was then tested by incubating CHO transfected and control cells with Alexa488-conjugated EGF (Fig. 10B, C). Staining with anti-HA antibody in combination with direct visualization of EGFAlexa488 (Fig. 10C) on control and transfected cells revealed co-localization of HA and EGFAlexa488 at membrane surface of one out of four transfected cells (arrow), but in none of control cells. Alternatively, pTWEEN*FRP3-HACD4* transfected cells (Fig. 10D) were incubated with a recombinant mouse EGF followed by the addition of the non-cleavable, cross-linker DTSSP. Staining with anti-EGF antibody (Fig. 10E) in combination with direct visualization of GFP showed that in this case virtually all transfected cells bind EGF in the extra-cellular space (Fig. 10F). A single FRP-3/GFP positive cell binding EGF on the surface is shown at high magnification in Fig. 10G–I. The higher number of FRP-3 expressing cells that bind EGF on the cell surface in the presence of the DTSSP cross-linker is a strong indication of a low affinity interaction.

## Discussion

The elucidation of the sequence of sFRP proteins immediately suggested their possible function. A highly conserved, cystein rich domain (CRD), presumed to bind Wnts, in the absence of a trans-membrane domain represents the structural requirement for a dominant negative molecule. It was thus suggested that sFRP-3 may sequester Wnts in the extra-cellular space and prevent binding to the Frizzled membrane receptors [8]. How this may occur in molecular terms is not yet completely understood but the recent elucidation of sFRP-3 crystal structure led to identify a Wnt-binding site in the CRDs exhibiting a conserved dimer interface that may be a feature of Wnt signaling [24]. Indeed, all the initial reports describing the biological effects of sFRP-3 in different developmental processes supported this hypothesis [8], [20], [25], [26]. However, it was recently reported that sFRP-3 unexpectedly increased osteoblast differentiation through a β-catenin-independent pathway in addition to its previously known function as a decoy receptor for Wnts [27].

The novel and unforeseen finding reported here is the antagonism between sFRP-3 and EGF. While studying the effects of sFRP-3 on mouse fibroblasts expressing Wnt-1, we noticed that sFRP-3 also inhibited proliferation of control fibroblasts that do not express Wnt-1 or any other Wnts at significant levels. We thus wondered whether sFRP-3 could exert its inhibitory effect by interfering with response to different growth factors. The results clearly indicated that sFRP-3 expressing cells did not proliferate in response to EGF while responding to the other growth factors exactly as their control counterparts. The specificity of the interaction between sFRP-3 and EGF was assessed further in another cell type, NIH-3T3 cells, where we could test a specific acute effect induced by these growth factors, i.e. the increase in [Ca^2+^]_i_ [15]. sFRP-3 was found to selectively counteract the effect of EGF on [Ca^2+^]_i_ increases, while not affecting the increase in [Ca^2+^]_i_ induced by FGF, PDGF or other stimuli that modify [Ca^2+^]_i_. This confirmed the specificity of interaction and also showed that the effect is probably direct since it acts in a period of few seconds, leading to inhibition of downstream transduction effectors. There is abundant evidence of multiple cross-talks between the EGF and the Wnt signaling pathways both in Drosophila and in vertebrates [28]–[34]. However this is the first evidence of a direct effect of sFRP-3 on EGF activity in mammalian cells.

Because of this novel observation, we investigated whether other biological activities of EGF could be neutralized by sFRP-3. Under certain conditions EGF induces morphological transformation and adhesion independent growth of mouse cells. When mouse 3T3 fibroblasts over-expressing the EGF receptor are exposed to EGF they acquire the ability to grow in soft agar and to form transformed foci on plastic. Both these activities were completely abrogated by sFRP-3 in a dose-dependent fashion. We then tested EGF-sFRP-3 interaction in a morphogenetic assay where EGF inhibits hair follicle development that occurs spontaneously in organ cultures of embryonic ectoderm. Strikingly, sFRP-3 reverted this inhibitory activity by causing the appearance of a normal number of follicles in the presence of EGF, without any effect on its own. Thus, all the biological activities of EGF that we have tested are blocked by sFRP-3 in a dose dependent way. However, EGF may induce synthesis of some Wnt in target tissues and thus the inhibition by sFRP-3 would be explained by its canonical action on EGF-induced Wnt genes. Several indirect evidence argue against this possibility: (i) sFRP-3 inhibits EGF-stimulated Ca2+ uptake in a time-frame that precludes induction of Wnt transcription, translation and secretion; (ii) in the Xenopus experiments, EGF injection alone did not cause axis duplication. Nevertheless, this possibility was formally ruled out by a detailed analysis of Wnt family genes expressed by 10T1/2 fibroblasts, ectoderm and somite explants which revealed that none of the 84 genes tested was induced by EGF.

On the other hand, the inhibition by sFRP-3 on EGF effects could be explained by their direct interaction or by sFRP-3 binding to EGF receptor.

As a matter of fact, EGF protein is expressed in the neuro-ectoderm and in the mesoderm contiguously to regions where the sFRP-3 messanger is expressed: these include a ventral area of the neural tube, the myotome and dermomyotome in somites and a proximal area of the developing limbs (see Fig. S2). Testing this hypothesis by classic knock out or morpholino loss of function experiments is complicated by the fact that the effects of either sFRP-3 or EGF cannot be analyzed separately from interaction with their primary ligands. In fact, blocking sFRP-3 by Xenopus morpholino injection experiments show a disorder in Wnt's pathways. The injected embryos present eye and fore brain disorder due to the lacking of antagonism (sFRP-3) to inhibit the posteriorizing effects of Wnt's signals (see Fig. S3), as already demonstrated in Xenopus and other vertebrates for several Wnt's antagonists [35]–[38]. However, in the Xenopus ectoderm, where Wnts are not known to be expressed, ablation of sFRP-3 caused a delay in the cement gland differentiation (see Fig. S4). This adhesive organ, that allows the Xenopus embryo to attach to objects in the water by secreting mucus, arise from the ectoderm that forms a pseudo-stratified columnar epithelium expressing cytokeratins by stage 28/29 NF [39]. Thus, the delay in ectoderm differentiation induced by ablation of sFRP-3 during the cement gland development suggest a role of sFRP-3 in regulating EGF-induced proliferation that maintains the ectoderm in an undifferentiated state.

Moreover, in gain of function experiments, we show that in the large majority of sFRP-3 treated embryos co-injection of EGF can restore a normal axis, whose elongation is blocked by sFRP-3. In the mouse embryo, where sFRP-3 affects axis elongation similarly to its effect in Xenopus [20], the concomitant transplacental delivery of both sFRP-3 and EGF (but not of FGF8) restored a normal embryo morphology in the majority of the embryos and also restored sFRP-3-dependent inhibition of myogenesis as it does *in vitro*.

All these data indicate a reciprocal interference between sFRP-3 and EGF in all the assays that we have used, both in Xenopus and in mouse. The most likely explanation for these phenomena is a physical interaction of the two proteins, possibly involving their CRD.

Examples of non canonical protein-protein interaction have been reported to play a significant if not a major role in tissue and organ morphogenesis. For example, the Cerberus protein functions as a multivalent growth factor that antagonizes Nodal, BMP and Wnt proteins by direct interaction in the extra-cellular space, via independent binding sites [40]. Also, Chordin antagonizes signaling by bone morphogenetic proteins (BMPs) by blocking binding to their receptors. Chordin, which is able to dorsalize mesoderm and to neuralize ectoderm, binds to BMP-4 with high affinity but not to activin or TGF-β1 [41]. Very recently it was shown that PDGFbb binds to the VEGF receptors and affects VEGF-dependent *in vitro* proliferation [42].

Therefore, this newly described interaction is not unprecedented but still raises questions on its possible significance during vertebrate embryogenesis. It should be noted that the binding affinity is low, which made it difficult to document specificity, but this fact has probably a significance. We propose that a low affinity binding for a relatively abundant molecule such as sFRP-3 may represent a way to prevent ligand diffusion outside of its domain of action.

It is important to underline that in Drosophila morphogenesis many fate choice decisions are dictated by a competition between the Wg and the EGFR signaling pathways. For example, cells in the Drosophila eye are resistant to apoptosis induced by activated Armadillo for a long period prior to the onset of cell death at the mid-pupal stage. This latency is due to EGF receptor (EGFR)/MAP kinase signaling since when it naturally ceases, the cells rapidly die. Thus, activated Armadillo is subject to a specific negative control by EGFR/Rolled MAP kinase signaling [43]. Furthermore, in the ventral epidermis Wingless signaling specifies smooth cells that produce naked cuticle, whereas the activation of the Drosophila epidermal growth factor receptor (DER) leads to the production of intercalating cells with cytoplasmic extrusions known as denticles. The DER pathway promotes denticle formation by activating *svb* expression that is known to induce, cell autonomously, cytoskeletal modifications producing the denticles [33]. Conversely, Wingless promotes the smooth cell fate through the transcriptional repression of *svb* by the bipartite nuclear factor Armadillo/dTcf. Moreover recent findings show the existence of a cortical region (cortical antihem) in which EGF family members and the secreted Wnt antagonist sFRP-2 are co-expressed [44].

The identification of a sFRP-3/EGF interaction in vertebrate development outlines a previously unknown developmental mechanism; it also opens a new area of investigation on the specific role of such interaction in the development of vertebrate neuroectoderm and mesoderm.

## Materials and Methods

### Cell culture and proliferation assay

C3H10T1/2 fibroblasts, Bosc23 packaging cells, 293FT and NIH 3T3 cells overexpressing the EFG receptor (clone 17, 4×10^5^ receptor/cells) were grown in Dulbecco's modified Eagle's medium (DMEM) (Gibco), supplemented with 10% fetal calf serum (FCS, Gibco). CHO cells were grown in α-MEM (Gibco) supplemented with 10% FCS.

To measure proliferation, 800–1200 cells/well were plated in 96-well plates and kept overnight in DMEM containing 2% horse serum, before shifting the cells to the same medium supplemented with the specific growth factor (day 0): EGF (100 ng/ml, Sigma), bFGF (10 ng/ml), PDGF-BB (0.4 nM) or HGF (20 ng/ml). The culture medium was changed every 48 hours. At different times, cells were fixed and stained in 0.1% crystal violet solution in 200 mM 4-morpholinoethanesulfonic acid, pH 6. Cell number was calculated from a calibration curve (cell number/OD 600 nm) done under the same conditions [45]. In another set of experiments 10^6^ C3H10T1/2 cells, wt or expressing Wnt1 or sFRP-3 (see below), and maintained in DMEM 10% FCS, were starved in DMEM serum free medium. After 24 serum was added again to the medium and cells analyzed for their DNA content by flow cytometry after Propidium Iodide staining, after 0, 18 and 20 h.

Conditioned medium (CM) was prepared as described [8]. Briefly, 293FT cells were transiently transfected with sFRP-3-HA plasmid and culture medium was collected from cells 5 days after transfection. For cell culture experiments CM was used undiluted (SFRP3 100%) or diluted 1∶2 with culture medium (SFRP3 50%), and additionated with growth factors prior to be added to cells.

### Western blot analysis

Western blot analysis was performed as described [46], using the following antibodies: tubulin B-5-1-2 (Zymed); HA (Babco); EGF (Sigma); AKT; P-AKT; MAPK; P-MAPK (Cell Signalling). Reactive proteins were detected with horseradish peroxidase-conjugated secondary antibodies and visualized with the enhanced chemioluminescence detection Kit (Amersham International PLC).

### Growth in soft agar and foci formation

Growth in soft agar and foci formation efficiency of NIH 3T3 cells clone 17 cells in the presence of increasing concentration of EGF (Calbiochem) in presence or absence of sFRP-3 conditioned medium were evaluated according to [47].

### [Ca^2+^]_i_ measurement

NIH 3T3 clone 17 cells were suspended in KRH medium [15] at pH 7.4 and loaded with the Ca^2+^-sensitive dye, fura-2, for 30 min at 25°C and kept at 37°C until use. The experiments were initiated by incubating cell aliquots (2×10^6^ cells in 750 µl) with 10 µl of medium conditioned either by sFRP-3-expressing or not expressing cells for 15 min at 37°C. Cells were subsequently centrifuged and resuspended in KRH supplemented with 250 µM sulfinpyrazone to prevent dye leakage. To measure the variations in the intracellular Ca^2+^ concentration ([Ca^2+^]_i_) induced by EGF, FGF, PDGF, UTP, ionomycin or thapsigargin, the samples were transferred to a thermostatted cuvette (37°C), maintained under continuous stirring, and analyzed in a Perkin Elmer LS-50B fluorimeter as described [15].

### EGF pathway activation

NIH 3T3 clone 17 cells were stimulated with EGF or bFGF in presence or absence of sFRP-3 conditioned medium for 30′ or 60′. Phosphorylation of AKT and MAPK was detected by western blot analysis using P-AKT and P-MAPK specific antibodies.

### Retrovirus preparation and viral infection

The mammalian retroviral vector pBABE-puro was used to construct and express full-length murine *sFRP-3* and *Wnt-1*. *sFRP-3* cDNA was generously provided by L. Leyns [8] and *Wnt-1* cDNA by J. Papkoff [48].

Recombinant retroviruses were generated by pEI (polyethylenimine Aldrich 40872-7) transfection of retroviral construct into Bosc23 packaging cells as previously described [49]. Two days later the viral supernatants were harvested, filtered and used to infect the cells for three hours in the presence of 8 µg/ml of polybrene (Sigma). 24 hr later the cultures were selected in 2 µg/ml of Puromycin (Sigma). Individual clones were isolated and expanded.

### RNA synthesis

Synthetic capped sFRP-3, EGF and FGF8 mRNAs were obtained from linearized pCS2m*FRZB*-HA, pCMV-SPORT6/*EGF* (IMAGE clone 4164289, from Open Biosystems) and pCS2*FGF8* [17] plasmids, using the mMESSAGE mMACHINE kit (Ambion), according to the instruction manual.

### Xenopus embryo manipulation and whole mount immunostaining

*Xenopus laevis* embryos were obtained by standard procedures and staged according to Nieuwkoop and Faber (NF) [50], cultured in Normal Amphibian Medium (NAM) at 24°C. The embryos were microinjected in one blastomere at 4-cell stage in Petri dishes coated with 1.5% noble agar and then cultured in NAM/10. For immunofluorescence and whole mount immunostaining embryos were treated according to [51]. For muscles labelling we used 12–101 monoclonal antibody [52] and for cytokeratins labelling we used Cytokeratins Wide Spectrum (Dako).

### Placenta injection of WOP cells

WOP (Without Origin of Polyoma DNA replication) cells were stably transfected with pCMV-SPORT6/*EGF* (IMAGE clone 4164289, from Open Biosystems), pcDNA3/sFrzb-3 [20] or pCS2*FGF8* [17] vectors with Fugene (Roche), following the manufacturer instructions. 5×10^5^ cells in 5 µl of PBS were injected into the maternal side of the placenta through the uterine wall, as previously described [20]. Usually, one uterine horn of the embryos at approximate age of E9.5 was injected, in order to avoid massive embryo abortion.

### Explants culture

Pre-somitic mesoderm explants from *Myf5a* embryos [53] at E9.5 dpc (20–24 somites) were cultured as previously described [54], [55]. After fixation in 4% paraformaldehyde, explants were analyzed for myosin heavy chain expression by immunofluorescence. Ectoderm explants were obtained from CD1 embryos at E13.5 as previously described [16]; after culture the explants were fixed in 4% paraformaldehyde and paraffin sectioned. Skin sections were stained with standard hematoxylin-eosin coloration and then analyzed for hair follicle differentiation under the microscope. For RT-PCR experiments, E9.5 somites and E13.5 CD1 embryonic skin explants were cultured for 24 h in Dulbecco's modified Eagle's medium (DMEM) (Gibco), supplemented with 2% horse serum (HS, Gibco) and 100 ng/ml EGF.

### Wnt-pathway PCR-array

RNA was extracted from 10T1/2 cells, E9.5 somites and E13.5 ectoderm explants 24 h after culture with or without EGF by using TRIzol (Invitrogen). Genomic DNA contamination was eliminated by Dnase treatment by using Rneasy Micro Kit (Qiagen) and RNA was retro-transcribed by RT^2^ First Strand Kit (Bioscience Corporation). Mouse Wnt Signaling Pathway RT2 Profiler PCR Array (http://www.superarray.com/rt_pcr_product/HTML/PAMM-043A.html) and RT2 Real-Timer SyBR Green/ROX PCR Mix were purchased from SuperArray Bioscience Corporation. PCR was performed on iCycler (Thermal Cycler Biorad), with the iQ detection system (Biorad). For data analysis the ΔΔCt method was used; for each gene fold-changes were calculated as difference in gene expression between control and EGF-treated samples. A positive value indicates gene up-regulation and a negative value indicates gene down-regulation. The formula used to calculate the relative gene expression level (2^ˆ^ (−Δ Ct)) is: ΔCt = Ct (GOI)−avg. (Ct (HKG)), where GOI is each gene of interest, and HKG are the housekeeping genes chosen for the “EGF-Control Gene”.

### Immunofluorescence

Immunofluorescence on cells and mesoderm explants was performed as previously described [20], using the following antibodies: HA (Babco), EGF (Sigma), MyHC [56]. Immunofluorescence on mouse cryostat sections was performed as previously reported [57] using the following antibodies: EGF (Sigma), nestin (DHSB).

### Production of sFRP-3CD4 chimeric protein

Construct for expression of CD4-anchored sFRP-3-HA protein was prepared as following. The *FRP3-HA* coding sequence was PCR-amplified from pCS2*mFRZB-HA* vector with a forward *EcoRI*-flanked primer and a reverse *XhoI*-flanked primer where the stop codon was replaced by an *AflII* site (5′-CTACTCGAGACATTTCAGGATCTTAAGTCGTACTGGCCGGG-3′). The FRP-HA PCR fragment was then cloned *EcoRI/XhoI* into pBSK vector. The trans-membrane domain of the hCD4, flanked by spacer sequences and containing its stop codon, was PCR-amplified from pCMV-SPORT6/*CD4* with a forward primer containing an *AflII* site (5′-CTGCTGGAATCCAACCTTAAGGTTCTGCCCACA TGGTCCA-3′) and a reverse *XhoI*-flanked primer (5′-CAGACTCGAGGGAGGCTGCAAGTGGG ATCTGCC-3′). The CD4 PCR product was *AflII*/*XhoI* double digested and then inserted into pBsFRP-3-HA vector, fused in frame to the COOH-terminus of FRP-HA. The *FRP3-HACD4* fragment was then cloned *EcoRI/XhoI* into pCS2 vector, downstream the CMV promoter, or *XbaI*/*XhoI* into pRRL.CMV.cPPT.hPGK.EGFP.WPRE vector [58], upstream the PGK*EGFP* cassette. We called these constructs pCS2*FRP3-HACD4* and pTWEEN*FRP3-HACD4*, respectively. Cell surface expression of CD4-anchored sFRP-3-HA protein was monitored by immunofluorescence staining with anti-HA antibody or live GFP expression.

### sFRP-3 protein *in vitro* purification

sFRP-3-HA protein was produced as previously described [46]. For protein purification, 10 ml of conditioned medium (CM) from 293FT cells transfected with sFRP-3-HA plasmid were incubated with anti-HA affinity matrix (Covance) for 2 hours at 4°C. After washing in PBS, biologically active sFRP-3-HA protein was purified by competitive elution with 1mg/ml HA peptide (Roche).

### *In vitro* binding, chemical cross-linking and Co-IP

1 µg of purified sFRP-3-HA protein was incubated with 1 µg of recEGF (Sigma) in PBS for 2 hours on a rocker at 4°C. 5 mM DTSSP cross-linker (Pierce) was then added to the sample and the reaction mixture was incubated for 1 hr at 4°C. Cross-linking reaction was quenched by the addition of 20 mM Tris pH 7.5, for 15 min. The cross-linked product was immunoprecipitated with an anti-HA affinity matrix (Covance) and then resolved on 4–12% gradient SDS-PAGE (Invitrogen) in non-reducing conditions. Immunoblot was performed as described above using the following antibodies: HA (Babco), EGF (Sigma). For dose-dependent binding experiment, 100 nM purified sFRP-3-HA (200 ng) or 100 nM of Ha peptide (Sigma) was incubated for 1 hr at 4°C with increasing amount of recEGF (1 mM, 2 mM, 4 mM and 6 mM, respectively), crosslinked and the complexes were then analysed by Western blot.

### EGF-binding assay

Heparin-immobilized acrylic beads (Sigma) were saturated overnight at 4°C with 5 µg of either recEGF (Sigma) or recFGF2 (Invitrogen) diluted in PBS 0.2% BSA. As negative control, heparin beads were incubated in PBS 0.5% BSA. To test whether sFRP-3 binds specifically to EGF, EGF-loaded beads were incubated for 2 hours at room temperature with increasing amount of sFRP-3-HA (5 µl, 10 µl, 20 µl and 50 µl of sFRP-3 conditioned medium), whereas FGF2-loaded and control beads were incubated with the highest amount of sFRP-3-HA. After 3 washing in PBS, beads were resuspended in 20 µl of lamely buffer, boiled and loaded onto a protein gel. Western blot analysis for HA or EGF was performed as described before.

### Binding affinity analysis

25 nM of EGFAlexa488 (Molecular Probes) was incubated with increasing amount (25 nM to 300 nM) of mouse recombinant sFRP-3 (R&D) in PBS, and then absorbance spectrum of Alexa488 was measured with a luminescence spectrometer LS50B (Perkin Elmer). The competition assay was performed by adding 250 nM of native EGF to 25 nM EGFAlexa488 prior to the addition of 100 nM sFRP-3. The absorbance spectrum of Alexa488 was measured as before.

## Supporting Information

Figure S1 sFRP-3 interferes with EGF signaling pathway AKT and MAPK phosphorylation in NIH-3T3 cells upon EGF stimulation is reduced in the presence of sFRP-3-CM (A), but not in the presence of bFGF (B).
(1.07 MB DOC)

Figure S2 Tissue distribution of sFRP-3 and EGF in the developing mammalian embryo (A,B,D,E,G,H) Immunofluorescence on cryostat transverse (A–C, G–H, 10× magnification) and parasagittal (D–E, 20× magnification) sections of mouse embryos at E10.5 using EGF (red) and nestin (green) antibodies. A, D, G show only expression of EGF and DAPI staining (blue), B, E, H show respectively the same sections as in A, D, G but EGF signal (red) is merged with the nestin signal (green). Arrow in H points to few cells in the limb expressing EGF. (C,F,I) In situ hybridization on cryostat transverse (C, I, 10× magnification) and parasagittal (F, 20× magnification) sections of mouse embryos at E10.5 using sFRP-3 specific riboprobe. C, F, I show sections that are alternating to those shown in A, D, G. Arrow in F points to cells in the myotome surrounded by sFRP-3 expressing cells. dn, dorsal neurons; drg, dorsal root ganglia; li, limb; mn, motorneurons; my, myotome; vz, ventricular zone.
(8.00 MB DOC)

Figure S3 sFRP-3 Moprholino Oligo's injection affects Wnt pathway. (A–C) Immunofluorescence on stage 28 NF Xenopus embryo sections against cytokeratins (red) and counterstained by 4′,6′-diamidino-2-phenylindole (DAPI) blue. (A) Normal wild type un-injected embryo presenting intense ketatinization on the cement gland, as revealed by cytokeratins red labeling. (B) sFRP3 morpholino (sFRP3-MO) injected embryo showing an almost devoid of cytokeratins red labeling on the adhesive organ. (C) sFRP3-MO/mRNA co-injected embryo revealing restoration of keratin differentiation in the cement gland. Inserts: high magnification of the cement gland. (D) Western blott analysis on crude protein extracts from stage 28 NF Xenopus embryos showing the decreasing of the cytokeratins production in the sFRP3-MO treated embryos and its restoration on co-injected sFRP3-MO/mRNA embryos. The filter was hybridized against citokeratins (Pan-Cyto) and against laminin as internal control.
(5.57 MB DOC)

Figure S4 sFRP-3 ablation affects cement gland differentiation. (A–C) Immunofluorescence on stage 28 NF Xenopus embryo sections against cytokeratins (red) and counterstained by 4′,6′-diamidino-2-phenylindole (DAPI) blue. (A) Normal wild type un-injected embryo presenting intense keratinization on the cement gland, as revealed by cytokeratins red labeling. (B) sFRP3 morpholino (sFRP3-MO) injected embryo showing an almost devoid of cytokeratins red labeling on the adhesive organ. (C) sFRP3-MO/mRNA co-injected embryo revealing restoration of keratin differentiation in the cement gland. Inserts: high magnification of the cement gland. (D) Western blot analysis on crude protein extracts from stage 28 NF Xenopus embryos showing the decreasing of the cytokeratins production in the sFRP3-MO treated embryos and its restoration on co-injected sFRP3-MO/mRNA embryos. The filter was hybridized against cytokeratins (Pan-Cyto) and against laminin as internal control.
(5.42 MB DOC)

Table S1 The table shows the percentage of cells in G0/G1, S and G2/M, at t0, t18 and t20, calculated using the CellQuest analysis software.
(0.03 MB DOC)

Table S2 Expression profile of mouse Wnts signaling pathway genes in control (CT) and EGF-treated (EGF) C3H10T1/2 cells. The relative expression level of each gene is given by the 2^ˆ^−ΔCt value (see Materials and Methods for formula calculation). Fold differences >2 or <2 between EGF and CT samples are outlined in green and red, respectively, in the “Fold-up or down regulation” column.
(0.19 MB DOC)
