# Supplementary figures and images for: Binding of sFRP-3 to EGF in the Extra-Cellular Space Affects Proliferation, Differentiation and Morphogenetic Events Regulated by the Two Molecules
Source: PLoS One. 2008 Jun 18;3(6):e2471. doi: 10.1371/journal.pone.0002471 (PMC2424011; doi:10.1371/journal.pone.0002471)

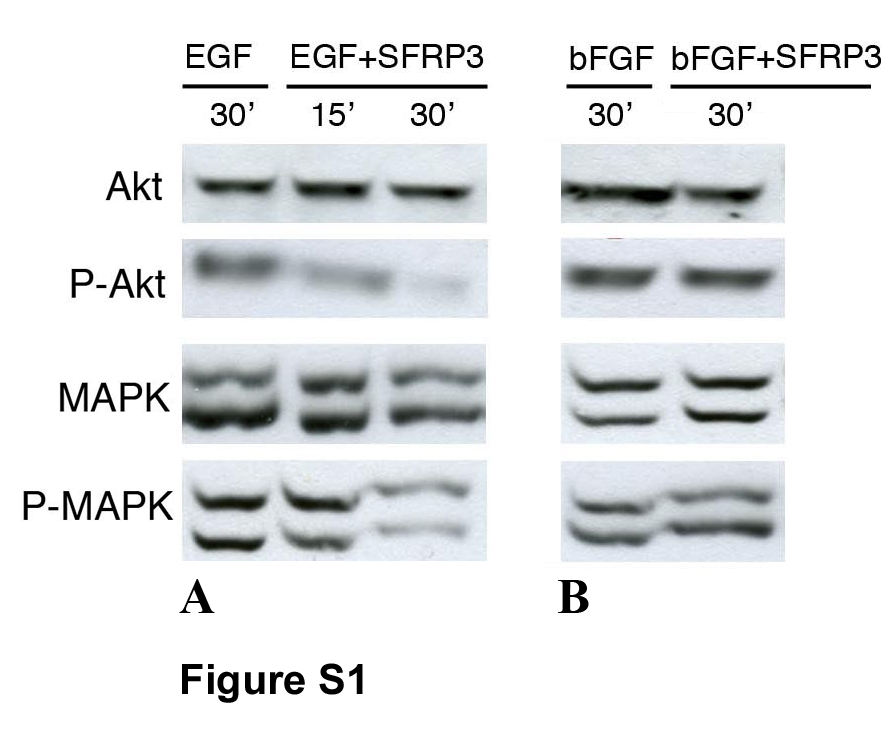

Supplement: Figure S1 — sFRP-3 interferes with EGF signaling pathway AKT and MAPK phosphorylation in NIH-3T3 cells upon EGF stimulation is reduced in the presence of sFRP-3-CM (A), but not in the presence of bFGF (B). (1.07 MB DOC) [file pone.0002471.s001.tif]

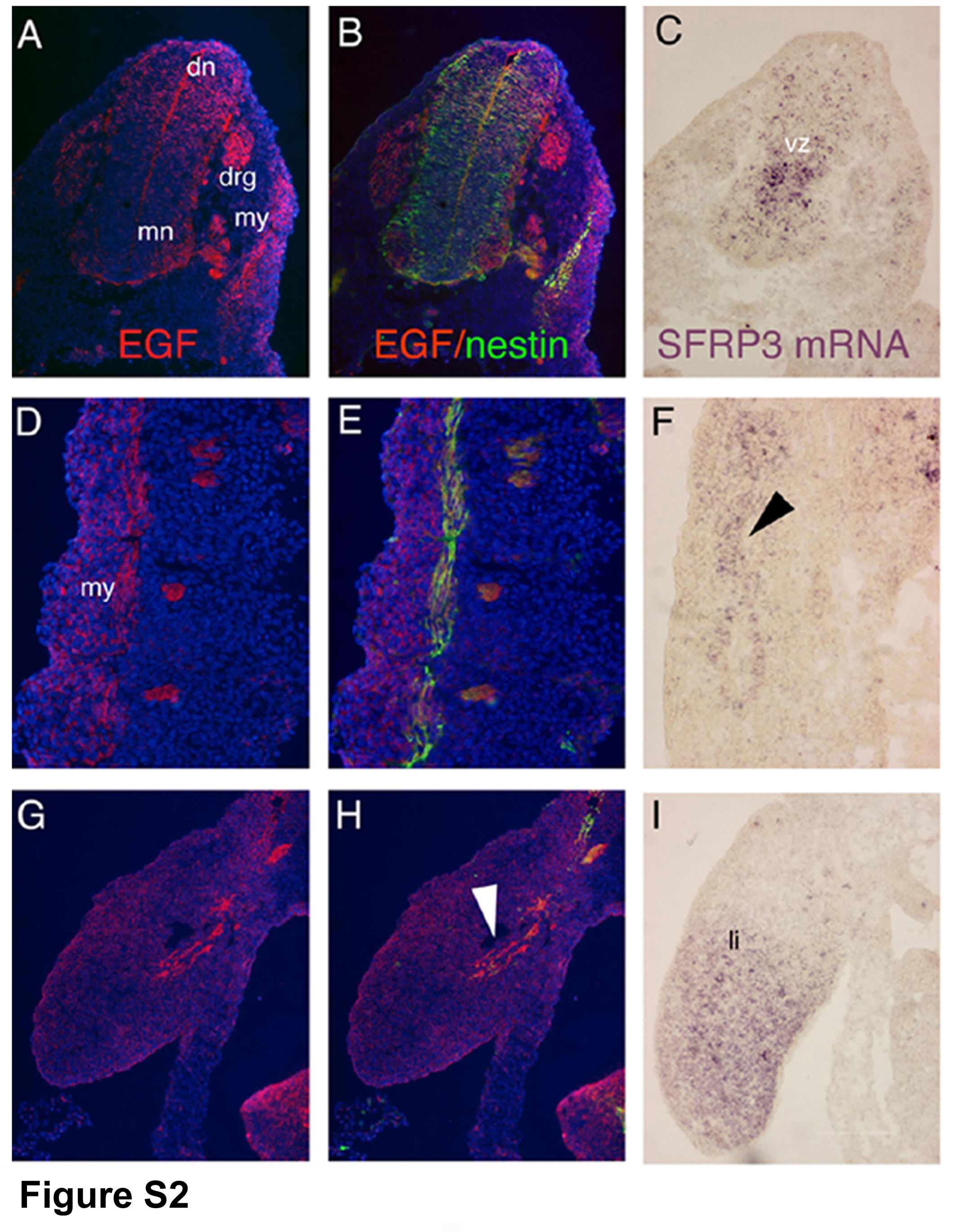

Supplement: Figure S2 — Tissue distribution of sFRP-3 and EGF in the developing mammalian embryo (A,B,D,E,G,H) Immunofluorescence on cryostat transverse (A–C, G–H, 10× magnification) and parasagittal (D–E, 20× magnification) sections of mouse embryos at E10.5 using EGF (red) and nestin (green) antibodies. A, D, G show only expression of EGF and DAPI staining (blue), B, E, H show respectively the same sections as in A, D, G but EGF signal (red) is merged with the nestin signal (green). Arrow in H points to few cells in the limb expressing EGF. (C,F,I) In situ hybridization on cryostat transverse (C, I, 10× magnification) and parasagittal (F, 20× magnification) sections of mouse embryos at E10.5 using sFRP-3 specific riboprobe. C, F, I show sections that are alternating to those shown in A, D, G. Arrow in F points to cells in the myotome surrounded by sFRP-3 expressing cells. dn, dorsal neurons; drg, dorsal root ganglia; li, limb; mn, motorneurons; my, myotome; vz, ventricular zone. (8.00 MB DOC) [file pone.0002471.s002.tif]

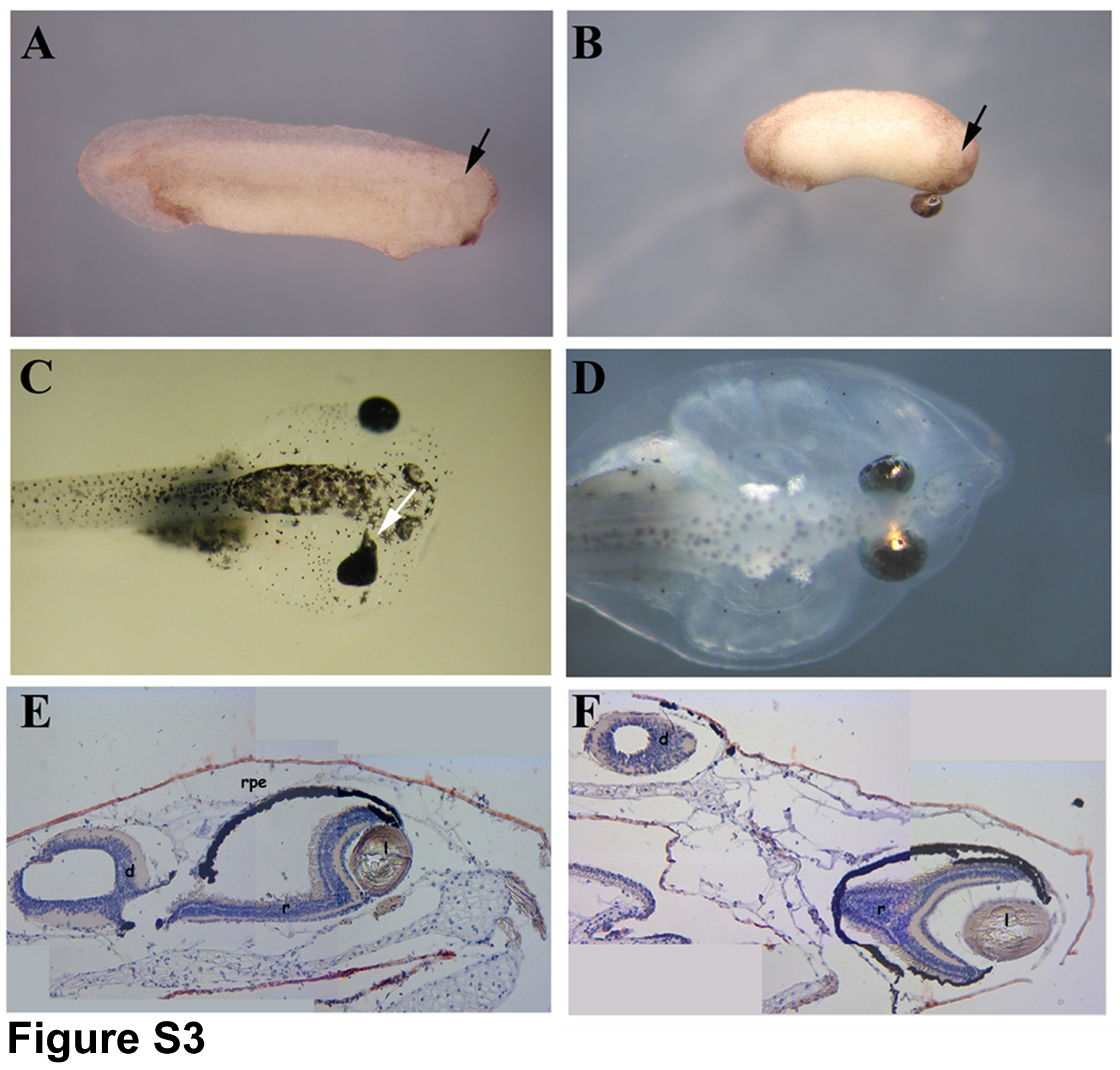

Supplement: Figure S3 — sFRP-3 Moprholino Oligo's injection affects Wnt pathway. (A–C) Immunofluorescence on stage 28 NF Xenopus embryo sections against cytokeratins (red) and counterstained by 4′,6′-diamidino-2-phenylindole (DAPI) blue. (A) Normal wild type un-injected embryo presenting intense ketatinization on the cement gland, as revealed by cytokeratins red labeling. (B) sFRP3 morpholino (sFRP3-MO) injected embryo showing an almost devoid of cytokeratins red labeling on the adhesive organ. (C) sFRP3-MO/mRNA co-injected embryo revealing restoration of keratin differentiation in the cement gland. Inserts: high magnification of the cement gland. (D) Western blott analysis on crude protein extracts from stage 28 NF Xenopus embryos showing the decreasing of the cytokeratins production in the sFRP3-MO treated embryos and its restoration on co-injected sFRP3-MO/mRNA embryos. The filter was hybridized against citokeratins (Pan-Cyto) and against laminin as internal control. (5.57 MB DOC) [file pone.0002471.s003.tif]

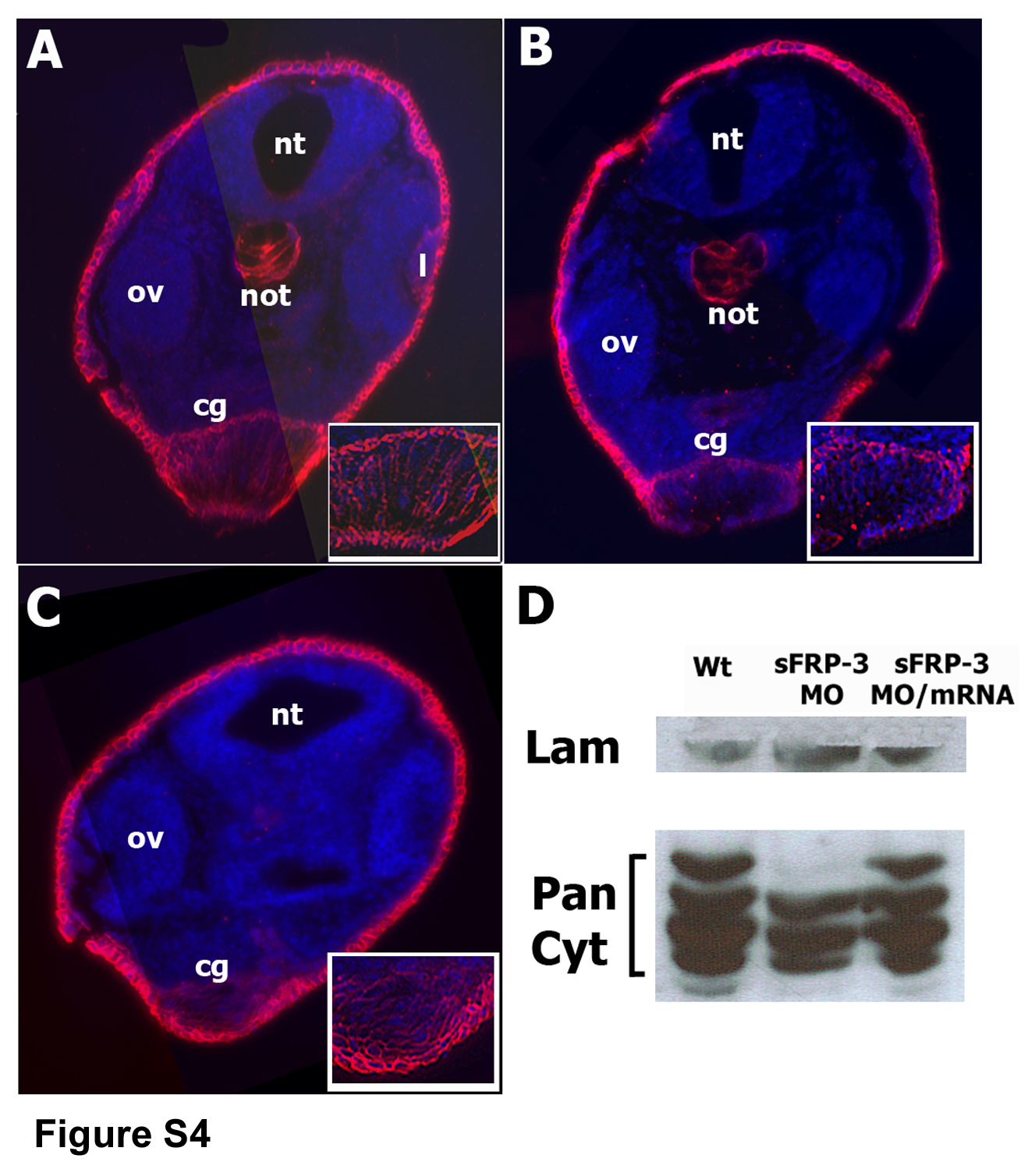

Supplement: Figure S4 — sFRP-3 ablation affects cement gland differentiation. (A–C) Immunofluorescence on stage 28 NF Xenopus embryo sections against cytokeratins (red) and counterstained by 4′,6′-diamidino-2-phenylindole (DAPI) blue. (A) Normal wild type un-injected embryo presenting intense keratinization on the cement gland, as revealed by cytokeratins red labeling. (B) sFRP3 morpholino (sFRP3-MO) injected embryo showing an almost devoid of cytokeratins red labeling on the adhesive organ. (C) sFRP3-MO/mRNA co-injected embryo revealing restoration of keratin differentiation in the cement gland. Inserts: high magnification of the cement gland. (D) Western blot analysis on crude protein extracts from stage 28 NF Xenopus embryos showing the decreasing of the cytokeratins production in the sFRP3-MO treated embryos and its restoration on co-injected sFRP3-MO/mRNA embryos. The filter was hybridized against cytokeratins (Pan-Cyto) and against laminin as internal control. (5.42 MB DOC) [file pone.0002471.s004.tif]
