# Supplementary material for: Binding of sFRP-3 to EGF in the Extra-Cellular Space Affects Proliferation, Differentiation and Morphogenetic Events Regulated by the Two Molecules
Source: PLoS One. 2008 Jun 18;3(6):e2471. doi: 10.1371/journal.pone.0002471 (PMC2424011; doi:10.1371/journal.pone.0002471)
Supplement: Table S1 — The table shows the percentage of cells in G0/G1, S and G2/M, at t0, t18 and t20, calculated using the CellQuest analysis software. (0.03 MB DOC) [file pone.0002471.s005.doc]

|  | C | | | SRP3 | | | Wnt1 | | |
| --- | --- | --- | --- | --- | --- | --- | --- | --- | --- |
| Time | Go/G1 | S | G2/M | Go/G1 | S | G2/M | Go/G1 | S | G2/M |
| 0h | 98,3±3,2 | 1,3±0,3 | 0,7±0,1 | 93,5±5,6 | 1,4±0,6 | 5,1±0,1 | 97,5±5,6 | 0,37±0,3 | 2,13±0,6 |
| 18h | 53,5±2,3 | 30,7±1,2 | 15,8±0,6 | 88,9±6,3 | 9,5±1,2 | 1,6±0,1 | 43,9±2,9 | 25,2±3,9 | 30,9±1,2 |
| 20h | 48,5±3,7 | 28,4±1,7 | 23,1±2,3 | 65,2±5,6 | 15,9±1,3 | 18,2±0,9 | 33,2±2,9 | 31,5±1,9 | 35,9±1,8 |

**Table S2.**
