# Supplementary material for: Binding of sFRP-3 to EGF in the Extra-Cellular Space Affects Proliferation, Differentiation and Morphogenetic Events Regulated by the Two Molecules
Source: PLoS One. 2008 Jun 18;3(6):e2471. doi: 10.1371/journal.pone.0002471 (PMC2424011; doi:10.1371/journal.pone.0002471)
Supplement: Table S2 — Expression profile of mouse Wnts signaling pathway genes in control (CT) and EGF-treated (EGF) C3H10T1/2 cells. The relative expression level of each gene is given by the 2ˆ−ΔCt value (see Materials and Methods for formula calculation). Fold differences >2 or <2 between EGF and CT samples are outlined in green and red, respectively, in the “Fold-up or down regulation” column. (0.19 MB DOC) [file pone.0002471.s006.doc]

| **Gene Symbol** | **Description** | **AVG ΔC_t_ (Ct(GOI) - Ave Ct (HKG))** | | **2^-ΔC_t_** | | **T-TEST** | **Fold Up- or Down-Regulation** |
| --- | --- | --- | --- | --- | --- | --- | --- |
|  |  | **EGF** | **CT** | **EGF Sample** | **CT**  **Sample** | **p value** | **EGF /CT** |
| Aes | Amino-terminal enhancer of split | 12,77 | 12,80 | 1,4E-04 | 1,4E-04 | 0,9670 | 1,02 |
| Apc | Adenomatosis polyposis coli | 5,66 | 5,51 | 2,0E-02 | 2,2E-02 | 0,4150 | -1,11 |
| Axin1 | Axin 1 | 4,57 | 4,56 | 4,2E-02 | 4,3E-02 | 0,9457 | -1,01 |
| Bcl9 | B-cell CLL/lymphoma 9 | 7,48 | 7,09 | 5,6E-03 | 7,4E-03 | 0,0440 | -1,31 |
| Btrc | b-transducin repeat containing protein | 6,70 | 6,63 | 9,6E-03 | 1,0E-02 | 0,5385 | -1,05 |
| Ctnnbip1 | Catenin beta interacting protein 1 | 5,52 | 5,62 | 2,2E-02 | 2,0E-02 | 0,0703 | 1,07 |
| Ccnd1 | Cyclin D1 | 2,01 | 2,50 | 2,5E-01 | 1,8E-01 | 0,0609 | 1,40 |
| Ccnd2 | Cyclin D2 | 2,32 | 2,06 | 2,0E-01 | 2,4E-01 | 0,2178 | -1,20 |
| Ccnd3 | Cyclin D3 | 3,17 | 3,16 | 1,1E-01 | 1,1E-01 | 0,9341 | -1,01 |
| Csnk1a1 | Casein kinase 1, alpha 1 | 2,24 | 2,14 | 2,1E-01 | 2,3E-01 | 0,6311 | -1,07 |
| Csnk1d | Casein kinase 1, delta | 3,26 | 3,25 | 1,0E-01 | 1,1E-01 | 0,9261 | -1,01 |
| Csnk2a1 | Casein kinase 2, alpha1polypeptide | 12,77 | 12,80 | 1,4E-04 | 1,4E-04 | 0,9670 | 1,02 |
| Ctbp1 | C-terminal binding protein 1 | 2,35 | 2,46 | 2,0E-01 | 1,8E-01 | 0,6820 | 1,08 |
| Ctbp2 | C-terminal binding protein 2 | 4,12 | 4,12 | 5,7E-02 | 5,8E-02 | 0,9584 | -1,00 |
| Ctnnb1 | Catenin, beta 1 | 1,63 | 1,41 | 3,2E-01 | 3,8E-01 | 0,4530 | -1,17 |
| Daam1 | Dishevelled associated activator of morphogenesis 1 | 7,07 | 6,33 | 7,4E-03 | 1,2E-02 | 0,0307 | -1,67 |
| Dixdc1 | DIX domain containing 1 | 5,80 | 5,82 | 1,8E-02 | 1,8E-02 | 0,9662 | 1,01 |
| Dkk1 | Dickkopf homolog 1 | 12,77 | 12,80 | 1,4E-04 | 1,4E-04 | 0,9670 | 1,02 |
| Dvl1 | Dishevelled, dsh homolog 1 | 5,65 | 5,23 | 2,0E-02 | 2,7E-02 | 0,0490 | -1,34 |
| Dvl2 | Dishevelled 2, dsh homolog | 4,83 | 5,05 | 3,5E-02 | 3,0E-02 | 0,1411 | 1,16 |
| Ep300 | E1A binding protein p300 | 4,50 | 4,47 | 4,4E-02 | 4,5E-02 | 0,6441 | -1,02 |
| Fbxw11 | F-box and WD-40 domain protein 11 | 4,93 | 4,87 | 3,3E-02 | 3,4E-02 | 0,4506 | -1,05 |
| Fbxw2 | F-box and WD-40 domain protein 2 | 3,42 | 3,42 | 9,3E-02 | 9,4E-02 | 0,9107 | -1,01 |
| Fbxw4 | F-box and WD-40 domain protein 4 | 5,41 | 5,11 | 2,3E-02 | 2,9E-02 | 0,0101 | -1,24 |
| Fgf4 | Fibroblast growth factor 4 | 12,77 | 12,80 | 1,4E-04 | 1,4E-04 | 0,9670 | 1,02 |
| Fosl1 | Fos-like antigen 1 | 3,48 | 4,80 | 8,9E-02 | 3,6E-02 | 0,0026 | **2,49** |
| Foxn1 | Forkhead box N1 | 12,00 | 11,96 | 2,4E-04 | 2,5E-04 | 0,9554 | -1,03 |
| Frat1 | Frequently rearranged in advanced T-cell lymphomas | 12,03 | 10,23 | 2,4E-04 | 8,3E-04 | 0,0213 | **-3,48** |
| Frzb | Frizzled-related protein | 12,77 | 12,80 | 1,4E-04 | 1,4E-04 | 0,9670 | 1,02 |
| Fshb | Follicle stimulating hormone beta | 12,77 | 12,80 | 1,4E-04 | 1,4E-04 | 0,9670 | 1,02 |
| Fzd1 | Frizzled homolog 1 | 1,58 | 1,44 | 3,3E-01 | 3,7E-01 | 0,6558 | -1,10 |
| Fzd2 | Frizzled homolog 2 | 5,57 | 5,15 | 2,1E-02 | 2,8E-02 | 0,1299 | -1,35 |
| Fzd3 | Frizzled homolog 3 | 10,20 | 10,09 | 8,5E-04 | 9,2E-04 | 0,7725 | -1,09 |
| Fzd4 | Frizzled homolog 4 | 6,39 | 5,55 | 1,2E-02 | 2,1E-02 | 0,0562 | -1,79 |
| Fzd5 | Frizzled homolog 5 | 5,37 | 5,07 | 2,4E-02 | 3,0E-02 | 0,0292 | -1,23 |
| Fzd6 | Frizzled homolog 6 | 9,24 | 9,26 | 1,6E-03 | 1,6E-03 | 0,9511 | 1,01 |
| Fzd7 | Frizzled homolog 7 | 4,20 | 3,76 | 5,4E-02 | 7,4E-02 | 0,4226 | -1,37 |
| Fzd8 | Frizzled homolog 8 | 5,62 | 4,33 | 2,0E-02 | 5,0E-02 | 0,0490 | **-2,45** |
| Gsk3b | Glycogen synthase kinase 3 beta | 6,13 | 6,10 | 1,4E-02 | 1,5E-02 | 0,2044 | -1,03 |
| Jun | Jun oncogene | 3,16 | 3,72 | 1,1E-01 | 7,6E-02 | 0,1119 | 1,47 |
| Kremen1 | Kringle containing transmembrane protein 1 | 4,22 | 4,32 | 5,4E-02 | 5,0E-02 | 0,6547 | 1,07 |
| Lef1 | Lymphoid enhancer binding factor 1 | 7,70 | 8,44 | 4,8E-03 | 2,9E-03 | 0,0521 | 1,66 |
| Lrp5 | Low density lipoprotein receptor-related protein 5 | 3,81 | 3,42 | 7,1E-02 | 9,3E-02 | 0,2875 | -1,31 |
| Lrp6 | Low density lipoprotein receptor-related protein 6 | 2,80 | 2,44 | 1,4E-01 | 1,8E-01 | 0,0458 | -1,29 |
| Myc | Myelocytomatosis oncogene | 3,98 | 4,25 | 6,3E-02 | 5,3E-02 | 0,2785 | 1,20 |
| Nkd1 | Naked cuticle 1 homolog | 7,91 | 5,79 | 4,2E-03 | 1,8E-02 | 0,0015 | **-4,34** |
| Nlk | Nemo like kinase | 6,83 | 6,33 | 8,8E-03 | 1,2E-02 | 0,1183 | -1,42 |
| Pitx2 | Paired-like homeodomain transcription factor 2 | 9,67 | 8,81 | 1,2E-03 | 2,2E-03 | 0,0386 | -1,82 |
| Porcn | Porcupine homolog | 7,41 | 6,58 | 5,9E-03 | 1,0E-02 | 0,1159 | -1,79 |
| Ppp2ca | Protein phosphatase 2 (formerly 2A), catalytic subunit, alpha isoform | 1,19 | 1,21 | 4,4E-01 | 4,3E-01 | 0,9050 | 1,01 |
| Ppp2r1a | Protein phosphatase 2 (formerly 2A), regulatory subunit A (PR 65), alpha isoform | 1,68 | 1,77 | 3,1E-01 | 2,9E-01 | 0,5791 | 1,06 |
| Ppp2r5d | Protein phosphatase 2, regulatory subunit B (B56), delta isoform | 3,22 | 3,32 | 1,1E-01 | 1,0E-01 | 0,4858 | 1,07 |
| Pygo1 | Pygopus 1 | 7,40 | 7,12 | 5,9E-03 | 7,2E-03 | 0,1554 | -1,21 |
| Rhou | Ras homolog gene family, member U | 6,12 | 5,55 | 1,4E-02 | 2,1E-02 | 0,0134 | -1,49 |
| Senp2 | SUMO/sentrin specific peptidase 2 | 5,20 | 5,17 | 2,7E-02 | 2,8E-02 | 0,8032 | -1,02 |
| Sfrp1 | Secreted frizzled-related protein 1 | 5,12 | 4,12 | 2,9E-02 | 5,8E-02 | 0,0050 | **-2,01** |
| Sfrp2 | Secreted frizzled-related protein 2 | 2,12 | 1,07 | 2,3E-01 | 4,8E-01 | 0,0156 | **-2,07** |
| Sfrp4 | Secreted frizzled-related protein 4 | 12,77 | 12,79 | 1,4E-04 | 1,4E-04 | 0,9732 | 1,01 |
| Slc9a3r1 | Solute carrier family 9, member 3 regulator 1 | 7,16 | 6,82 | 7,0E-03 | 8,9E-03 | 0,5374 | -1,27 |
| Sox17 | SRY-box containing gene 17 | 12,77 | 12,80 | 1,4E-04 | 1,4E-04 | 0,9670 | 1,02 |
| T | Brachyury | 12,77 | 12,80 | 1,4E-04 | 1,4E-04 | 0,9670 | 1,02 |
| Tcf3 | Transcription factor 3 | 5,06 | 4,86 | 3,0E-02 | 3,5E-02 | 0,4121 | -1,16 |
| Tcf7 | Transcription factor 7, T-cell specific | 6,60 | 6,52 | 1,0E-02 | 1,1E-02 | 0,3786 | -1,06 |
| Tle1 | Transducin-like enhancer of split 1, homolog of Drosophila E(spl) | 5,00 | 5,16 | 3,1E-02 | 2,8E-02 | 0,0059 | 1,11 |
| Tle2 | Transducin-like enhancer of split 2, homolog of Drosophila E(spl) | 8,33 | 7,64 | 3,1E-03 | 5,0E-03 | 0,1033 | -1,62 |
| Wif1 | Wnt inhibitory factor 1 | 12,77 | 12,80 | 1,4E-04 | 1,4E-04 | 0,9670 | 1,02 |
| Wisp1 | WNT1 inducible signaling pathway protein 1 | 2,27 | 1,76 | 2,1E-01 | 3,0E-01 | 0,0161 | -1,43 |
| Wnt1 | Wingless-related MMTV integration site 1 | 12,77 | 12,80 | 1,4E-04 | 1,4E-04 | 0,9670 | 1,02 |
| Wnt10a | Wingless related MMTV integration site 10a | 12,77 | 12,80 | 1,4E-04 | 1,4E-04 | 0,9670 | 1,02 |
| Wnt11 | Wingless-related MMTV integration site 11 | 12,77 | 12,80 | 1,4E-04 | 1,4E-04 | 0,9670 | 1,02 |
| Wnt16 | Wingless-related MMTV integration site 16 | 11,19 | 10,28 | 4,3E-04 | 8,0E-04 | 0,2845 | -1,88 |
| Wnt2 | Wingless-related MMTV integration site 2 | 10,98 | 10,64 | 5,0E-04 | 6,3E-04 | 0,5866 | -1,26 |
| Wnt2b | Wingless related MMTV integration site 2b | 9,06 | 9,12 | 1,9E-03 | 1,8E-03 | 0,8628 | 1,04 |
| Wnt3 | Wingless-related MMTV integration site 3 | 12,77 | 12,80 | 1,4E-04 | 1,4E-04 | 0,9670 | 1,02 |
| Wnt3a | Wingless-related MMTV integration site 3A | 12,77 | 12,80 | 1,4E-04 | 1,4E-04 | 0,9670 | 1,02 |
| Wnt4 | Wingless-related MMTV integration site 4 | 7,36 | 6,90 | 6,1E-03 | 8,4E-03 | 0,3489 | -1,38 |
| Wnt5a | Wingless-related MMTV integration site 5A | 8,38 | 7,36 | 3,0E-03 | 6,1E-03 | 0,0951 | **-2,04** |
| Wnt5b | Wingless-related MMTV integration site 5B | 8,92 | 8,18 | 2,1E-03 | 3,5E-03 | 0,1074 | -1,67 |
| Wnt6 | Wingless-related MMTV integration site 6 | 12,77 | 12,80 | 1,4E-04 | 1,4E-04 | 0,9670 | 1,02 |
| Wnt7a | Wingless-related MMTV integration site 7A | 12,77 | 12,80 | 1,4E-04 | 1,4E-04 | 0,9670 | 1,02 |
| Wnt7b | Wingless-related MMTV integration site 7B | 12,77 | 12,80 | 1,4E-04 | 1,4E-04 | 0,9670 | 1,02 |
| Wnt8a | Wingless-related MMTV integration site 8A | 12,77 | 12,80 | 1,4E-04 | 1,4E-04 | 0,9670 | 1,02 |
| Wnt8b | Wingless related MMTV integration site 8b | 12,77 | 12,80 | 1,4E-04 | 1,4E-04 | 0,9670 | 1,02 |
| Wnt9a | Wingless-type MMTV integration site 9A | 10,00 | 10,07 | 9,7E-04 | 9,3E-04 | 0,8769 | 1,05 |
| Hsp90ab1 | Heat shock protein 90kDa alpha, class B member 1 | -1,30 | -1,27 | 2,5E+00 | 2,4E+00 | 0,8248 | 1,02 |
| Gapdh | Glyceraldehyde-3-phosphate dehydrogenase | -0,95 | -0,72 | 1,9E+00 | 1,6E+00 | 0,1360 | 1,17 |
| Actb | Actin, beta, cytoplasmic | -3,89 | -4,00 | 1,5E+01 | 1,6E+01 | 0,5642 | -1,08 |

**Table S1.**
